# Novel selective glucocorticoid receptor modulator GRM-01 demonstrates dissociation of anti-inflammatory effects from adverse effects on glucose and bone metabolism

**DOI:** 10.3389/fphar.2025.1542351

**Published:** 2025-03-05

**Authors:** Florian Jakob, Stephanie Hennen, Michael Gautrois, Feras Khalil, Andrew Lockhart

**Affiliations:** Research and Development, Grünenthal GmbH, Aachen, Germany

**Keywords:** anti-inflammatory agents, glucocorticoids, glucocorticoid receptor modulator, gluconeogenesis, inflammation, osteoblasts, prednisolone

## Abstract

**Introduction:**

The development of selective GR agonist and modulators (SEGRAMs) aimed to minimize the adverse effects of chronic glucocorticoid treatment (e.g., hyperglycemia and osteoporosis) by separating the transactivation and transrepression activities of the glucocorticoid receptor (GR). Herein we report the pharmacologic profile of clinical candidate GRM-01, a novel, orally available, non-steroidal SEGRAM.

**Methods:**

*In vitro* GR, progesterone receptor (PR), and mineralocorticoid receptor (MR) binding and reporter gene assays were conducted to determine GRM-01 potency and selectivity. Anti-inflammatory effects were investigated *in vitro* using functional assays in rat and human whole blood, human lung cells, and primary fibroblast-like synoviocytes from human donors with rheumatoid arthritis. *In vitro* assays measured tyrosine aminotransferase [TAT] activity in human hepatocytes and osteoprotegerin release from human osteoblasts as markers of glucose and bone metabolism, respectively. *In vivo* studies examined the effect of GRM-01 on biomarkers in a rat model of inflammation and on cortisol levels in Cynomolgus monkeys. Animal pharmacokinetics (PK) for GRM-01 were determined and used to predict its human PK.

**Results:**

GRM-01 is a potent and selective ligand of human GR versus human PR and MR (inhibition constant = 12 vs. 3,700 and >10,000 nM, respectively). GRM-01 displayed partial induction (transactivation) at the GR (half-maximal effective concentration [EC_50_] = 60.2 nM, efficacy 31.8%) versus prednisolone (EC_50_ = 24.3 nM, efficacy 80.5%). GRM-01 demonstrated anti-inflammatory efficacy, inhibiting tumor necrosis factor-α and interferon-γ release in whole blood assays, and interleukin-6 release in cellular assays. GRM-01 weakly increased TAT activity in HepG2 cells (efficacy 14.0% vs. 92.4% with prednisolone) and partially inhibited osteoprotegerin release in MG-63 cells (by 58% vs. 100%). *In vivo*, GRM-01 dose-dependently reduced rat ankle swelling, had anti-nociceptive effects, and did not increase blood glucose. In Cynomolgus monkeys, GRM-01 dose-dependently reduced plasma cortisol. Animal PK found that GRM-01 had high oral bioavailability, generally low clearance, and good tissue partitioning. The predicted human total plasma clearance of GRM-01 was 0.25 mL/min/kg, volume of distribution 2.124 L/kg, and half-life ∼98 h.

**Conclusion:**

GRM-01 displays a favorable preclinical pharmacologic profile consistent with a SEGRAM, and based on this is currently in Phase 1 development.

## Introduction

Exogenous (synthetic) glucocorticoids such as prednisolone or dexamethasone are an important class of immunosuppressive drug used widely in a variety of allergic and inflammatory conditions including rheumatoid arthritis and asthma, and for the treatment of various blood cancers and prevention of transplant rejection (Buttgereit, 2020; Sundahl et al., 2015; Williams, 2018).

Glucocorticoids bind to glucocorticoid receptors (GRs) causing a conformational change in the receptor; the receptor then moves into the nucleus to bind to glucocorticoid response elements (GREs), thereby stimulating or suppressing gene transcription (transactivation or transrepression, respectively) (Williams, 2018). Additionally, glucocorticoids have non-genomic effects, including, for example, reducing airway smooth muscle tone, modulating intracellular calcium homeostasis, and inducing reactive oxygen species generation and endothelial nitric oxide species activity (Panettieri et al., 2019). GR transrepression of inflammatory and immune genes, such as those for cytokines and chemokines, is a key mechanism of the anti-inflammatory effects of glucocorticoids (Sundahl et al., 2015). However, due to the ubiquitous expression of the GR in vertebrate cells and consequent direct or indirect regulation of thousands of genes (Mao et al., 2023), glucocorticoids also cause a wide array of serious adverse effects that limit their clinical utility, particularly when long-term and/or high-dose therapy is needed (Baker, 2021). These adverse effects are mediated, by either transactivation (e.g., diabetes), transrepression (e.g., hypothalamic-pituitary-adrenal [HPA] axis suppression) or both transactivation and transrepression (e.g., osteoporosis) (Buttgereit et al., 2005).

Hyperglycemia and the development of type 2 diabetes mellitus, and bone adverse effects (Li and Cummins, 2022; van Staa, 2006), are among the most concerning glucocorticoid-associated adverse effects for patients and for clinicians to manage. In the liver, glucocorticoids directly upregulate enzymes in the gluconeogenesis pathway (stimulating hepatic glucose production) and promote hepatic insulin resistance, together leading to hyperglycemia (Li and Cummins, 2022). In bone, glucocorticoids reduce bone formation via inhibition of osteoblast differentiation and increase osteocyte apoptosis, which contributes to reduced bone mineral density (BMD) and altered bone architecture and quality, and a consequent increase in the risk of fractures (van Staa, 2006). Specifically, glucocorticoids inhibit the production of osteoprotegerin (OPG) by osteoblasts, resulting in an increase in osteoclastogenesis (Hofbauer et al., 1999).

The therapeutic approach to improving the benefit-to-risk ratio of glucocorticoids is to use the lowest possible dose for as short a duration as possible (Baker, 2021), but this can limit their therapeutic utility, and neglects addressing the mechanistic cause of the adverse effects associated with these agents.

Development of new drugs to overcome these adverse effects has taken several routes. One strategy has focused on keeping the steroid molecular skeleton, but with an altered structure in an attempt to maintain therapeutic effect with fewer corticosteroid-associated adverse effects. An example of this is the non-selective, dissociated steroid vamorolone, which binds the GR and mineralocorticoid receptor (MR), but unlike prednisolone, is (i) an MR antagonist, and (ii) induces less GRE-mediated gene transcription (meaning less transactivation) (Damsker et al., 2019; Keam, 2024). An alternative strategy is to move away from the steroidal skeleton towards novel non-steroidal selective therapeutics that demonstrate greater selectivity of effects at the GR, thus aiming for stronger separation between the two main modes of GR activity; such drugs would selectively bind to the GR receptor and retain full transrepression (anti-inflammatory) efficacy but have only partial transactivation (adverse) activity (Mao et al., 2023). One such class of drugs is the non-steroidal selective GR agonist and modulators (SEGRAMs) (Buttgereit et al., 2019; Hegelund Myrbäck et al., 2020; Hu et al., 2011; Ripa et al., 2018), which, being GR agonists, have been assigned the stem “-corat” (World Health Organization Inn Programme, 2024). To date, a small number of these drugs have entered clinical development (e.g., fosdagrocorat (PF-04171327) (Buttgereit et al., 2019); mizacorat (AZD9567) (van Laar et al., 2023)) but none have entered pivotal trials or been submitted for regulatory approval.

GR modulator-01 (GRM-01) is a novel SEGRAM, developed by Grünenthal GmbH and we report here for the first time the preclinical *in vitro* and *in vivo* pharmacologic effects of the drug. Data derived from a translationally-relevant preclinical model suggest that GRM-01 has a novel pharmacologic profile, characterized by separation of transrepression from transactivation, combined with good oral bioavailability and *in vivo* potency.

## Materials and methods

### Compounds, reagents and test conditions

GRM-01 was manufactured by Grünenthal GmbH (Aachen, Germany). Prednisolone, aldosterone, beclomethasone, progesterone, and fludrocortisone were supplied by Sigma-Aldrich (St Louis, MO, United States). Stock solutions of test and control compounds were prepared using dimethyl sulfoxide (DMSO). All solvents and commercial reagents were sourced commercially and were of laboratory grade.

All *in vitro* experiments were conducted at 37°C in 5% CO_2_ atmosphere, and at 95% humidity (hereafter referred to as ‘standard’ conditions), unless otherwise specified.

### *In vitro* pharmacology

#### Nuclear hormone receptor binding assays

GRM-01 and prednisolone affinity for nuclear hormone receptors was assessed in binding assays conducted by Eurofins Cerep (Celle l’Evescault, France) and Eurofins Panlabs Discovery Services Taiwan Ltd., (Taipei, Taiwan). The affinity of GRM-01 for the human GR endogenously expressed in human B lymphoblast IM-9 cell line was determined in supernatants from lysed IM-9 cells using the radioligand ^3^H-dexamethasone (final concentration 1.5 nM) as described by Clark et al. (Clark et al., 1996), and affinity for the human progesterone receptor (PR) was determined in adherent human epithelial breast carcinoma T47D cell line using the radioligand ^3^H-progesterone (final concentration 0.5 nM) as described by Sarup et al. (Sarup et al., 1988). Scintillation counting of bound radioligand was conducted after 6 and 20 h of incubation (at 4°C), respectively. Affinity of GRM-01 for the human MR, which was obtained from insect Sf9 cells recombinantly overexpressing human MR, was determined based on the inhibition of binding of the radioligand ^3^H-aldosterone (final concentration of 0.4 nM). Scintillation counting of bound radioligand was conducted after 20 h of incubation at 4°C.

In these assays, competition for binding at the human GR, PR, and MR between GRM-01 or prednisolone and the radioligands ^3^H-dexamethasone 1.5 nM, ^3^H-progesterone 0.5 nM, and ^3^H-aldosterone 0.4nM, respectively, was expressed as half-maximal inhibitory concentration (IC_50_) and inhibition constant (K_i_) values (Supplementary Material). For the comparison of binding to the GR and PR by GRM-01 and by prednisolone, IC_50_ values were determined by non-linear regression analysis of competition curves using software developed at Cerep (Hill Software) and validated by comparison with data generated by the commercially-available software SigmaPlot^®^ version 4.0 for Windows^®^ (SPSS Inc., Chicago, IL, United States), and K_i_ values were calculated using the Cheng Prusoff equation (Cheng and Prusoff, 1973) (see Supplementary Material for further detail). The same statistical methods were used for the MR binding assays, but using MathIQ™ software (ID Business Solutions Ltd., Woking, United Kingdom).

#### Nuclear hormone receptor reporter gene assays

GRM-01 induction of human GR, PR, and MR transactivation was tested in reporter gene assays, conducted by Axxam S.p.A (Bresso, Milan, Italy), in a Chinese hamster ovary (CHO) cell line (Art.-No. ACC-110, Leibniz Institute DSMZ - German Collection of Microorganisms and Cell Cultures GmbH, Braunschweig, Germany) overexpressing human GR, PR, or MR ligand binding domain (LBD) fused to the GAL4 DNA binding domain (LBD-DBD). To create the CHO-Gal4/GR, CHO-Gal4/PR, and CHO-Gal4/MR reporter cell lines, first CHO cells were stably transfected with a GAL4-UAS-luciferase reporter construct. Then, the LBD of the GR, PR, or MR (GR Gene ID: 2908, PR Gene ID: 5241, MR Gene ID: 4306, respectively) was cloned into pIRES2-EGFP-GAL4 containing the DBD of GAL4 from pFA-AT2, and then co-transfected into the luciferase-reporter construct-containing CHO cells. These reporter cell lines express the respective nuclear hormone receptor LBD-DBD fusion proteins that control luciferase expression of a GAL4-upstream activation sequence (UAS)-luciferase reporter construct, allowing for detection of ligand- or compound-induced transactivation at the GR, PR, or MR by measuring luciferase activity.

Cryo-preserved CHO-Gal4/GR, CHO-Gal4/PR, and CHO-Gal4/MR cells were plated in medium at 7,500 cells/well in 384-well assay plates (25 μL/well), and incubated for 24 h. The medium was removed and 30 μL assay buffer plus 10 μL of serially-diluted compound added per well for a final assay concentration range from 0.003 μM to 10 μM in 0.5% DMSO (four replicates per concentration). Control wells contained beclomethasone, progesterone, or fludrocortisone to a final assay concentration range from 0.0001 nM to 1 μM (in 0.5% DMSO), serving as reference agonists at the GR, PR, and MR, respectively. Following a 4-h incubation, cells were lysed and luminescence measured using the EMCCD camera FLIPR®Tetra (Molecular Devices, Silicon Valley, CA, United States).

Transactivation (i.e., gene activation) was expressed as a percentage of the reference agonist activity (Supplementary Material), using Genedata Screener^®^ v15.0.5 software (Basel, Switzerland). The EC_50_ values (the concentration that produces half-maximal efficacy) and Hill coefficients were determined by non-linear regression analysis of the competition curves with constraining Hill slope to 1 (in GraphPad Prism v7.03 [GraphPad Software, Boston, MA, United States]).

#### Anti-inflammatory profile in functional assays

The effect of GRM-01 on cytokine release was examined in rat and human whole blood, human cell lines, and human primary cells.

##### Inhibition of lipopolysaccharide (LPS)-induced tumor necrosis factor-alpha (TNF-α) release in rat whole blood

Arterial blood was collected from male Sprague-Dawley rats (180–250 g; Janvier Labs, Le Genest-Saint-Isle, France) in heparin syringes. The blood was added at 120 μL/well to a 96-well plate plus 15 μL/well LPS and 15 μL/well of each GRM-01 or prednisolone dilution (Supplementary Material), in duplicate, for a final assay concentration of 0.038 nM–10 μM in 0.1% DMSO, and incubated for 18 h in standard conditions. Plasma was collected via centrifugation and TNF-α measured using a TNFalpha Kit (PerkinElmer, Waltham, MA, United States), according to manufacturers’ instructions, and a microplate reader (EnVision Multilabel Reader^®^, PerkinElmer; Supplementary Material). The IC_50_ values for inhibition of TNF-α release were calculated by fitting concentration response curves to a four parameter nonlinear regression model using ActivityBase (XLfit^®^ software; ID Business Solutions Ltd.). TNF-α values obtained with medium plus LPS were defined as 0% inhibition and values obtained with medium in the absence of LPS as 100% inhibition. In addition, IC_50,free_ values were determined by correcting the IC_50_ values for plasma protein binding (PPB); see the Supplementary Material for the calculation. GRM-01 PPB values had been determined in a prior analysis (data on file).

##### Inhibition of LPS-induced interferon-γ (IFN-γ) release in human whole blood

This assay was conducted by Synexa Life Sciences BV in Cape Town, South Africa. Human whole blood (37.5 mL) collected from ten healthy volunteers in heparin-coated tubes was plated to 96-well plates (2 mL/well) containing 2 µL of GRM-01 or prednisolone 0.0096–30 mM solution (Supplementary Material), and incubated for 1 h in standard conditions. Appropriate control wells were included (2 μL DMSO ± LPS). Subsequently, 1 mL of whole blood solution from each well was incubated at 37°C for 24 h in LPS-containing culture tubes, where final test compound concentrations were 0.0032, 0.016, 0.08, 0.4, 2, and 10 μM. Culture supernatant was harvested using the Seraplas^®^ V11 valve filter (Sarstedt, Nümbrecht, Germany) and IFN-γ determined using validated methods with the V-PLEX assay (V-PLEX Proinflammatory Panel 1 Human Kit, Meso Scale Discovery, Rockville, MD, United States), as shown in Supplementary Table S1. The IC_50_ value for GRM-01 inhibition of LPS-induced IFN-γ release was calculated using a three-parameter non-linear regression (GraphPad PRISM software v8.4.1, Graphpad Software Inc.). If IC_50_ values so derived were above or below the tested concentration range, they were not reported due to the non-reliable curve fit. IC_50_ values corrected for PPB were also determined, giving IC_50,free_ values (using the same formula as for the TNF-α assay in rat whole blood).

##### Inhibition of interleukin-1β (IL-1β)–induced IL-6 release in A549 cells

Cells from the human lung epithelial cell line A549 (CCL-185, ATCC) were seeded in 96-well plates at a density of 30,0000 cells/well in F12K Nutrient Mix (Gibco™, ThermoFisher Scientific, Waltham, MA, United States) with 10% fetal calf serum (FCS), and cultured for 24 h in standard conditions. Before stimulation with GRM-01 or prednisolone, cells were incubated for 4 h in FCS-free F12K nutrient (150 μL/well). Then, 50 μL each of GRM-01 or prednisolone were added to wells (see Supplementary Material for solution preparation), in triplicate, resulting in a final assay concentration range of 2 μM to 0.0256 nM in 0.125% DMSO. After 30 min’ incubation, human IL-1β (1 ng/mL final concentration) was added at 50 µL/well, followed by a 20 h-incubation in standard conditions. Human IL-6 was measured in the collected supernatant using the human AlphaLISA^®^ immunoassay kit (PerkinElmer; Supplementary Material). Percentage inhibition of IL-6 release and IC_50_ values were calculated as described previously for TNF-α inhibition in rat whole blood, except 0% inhibition was defined as IL-6 values obtained with medium in the presence of IL-1β (1 ng/mL) and 100% inhibition as the values obtained in the presence of IL-1β (1 ng/mL) and 1 μM dexamethasone.

##### Inhibition of TNF-α–induced IL-6 release by primary fibroblast-like synoviocytes (FLS) from patients with rheumatoid arthritis

FLS were selected for this assay because these cells produce inflammatory cytokines that are involved in the inflammation and joint destruction seen in rheumatoid arthritis (Matsuda et al., 2023). In this assay, conducted by BioIVT (Hertfordshire, United Kingdom), primary FLS were obtained from three donors with rheumatoid arthritis and seeded at passage 4 in FGM™-2 fibroblast growth medium (Lonza) in 96-well plates at a density of 10,0000 cells/well, and incubated overnight in standard conditions. The cells were treated with GRM-01 or prednisolone (triplicate wells per concentration; Supplementary Material) and TNF-α, which was added at a previously-determined 90% maximal effective concentration (EC_90_; 1 ng/mL for FLS from donor 1, 5 ng/mL for FLS from donor 2, and 4 ng/mL for FLS from donor 3), and incubated for 24 h in standard conditions. Negative controls were TNF-α alone and culture medium alone, and vehicle control was DMSO (0.1%). IL-6 was measured in collected supernatant using an enzyme-linked immunosorbent assay (ELISA, R&D Systems Minneapolis, MN, United States) according to the manufacturer’s instructions. Percentage inhibition of IL-6 release by GRM-01 and prednisolone was calculated (Supplementary Material), and IC_50_ values and percentage efficacy (maximum percent inhibition according to curve fitting) determined in GraphPad Prism^®^ v7.03 (GraphPad Software Inc.) using a 4-parameter logistic nonlinear regression analysis with constraining Hill slope to 1.

#### Effect on tyrosine aminotransferase (TAT) activity in human hepatocyte cell line

TAT is a key enzyme in gluconeogenesis under the control of glucocorticoids and their interaction with the GR (Panin and Usynin, 2008), where induction of TAT expression is mediated through GR transactivation. This assay examined the effect of GRM-01 on the induction of TAT enzyme expression, and consequently on the gluconeogenesis pathway, and was conducted by Axxam S.p.A. (Bresso, Milan, Italy) in a hepatocyte carcinoma biopsy-derived cell line (HepG2, ECACC, Salisbury, United Kingdom), since the liver is the primary site for gluconeogenesis (Hatting et al., 2018). The impact of glucocorticoids on TAT in HepG2 cells has been shown to be blocked by the selective GR antagonist mifepristone (RU486), demonstrating that the mechanism involves GR and not MR (Hu et al., 2017).

HepG2 cells were seeded at 20,000 cells/well in an Eagles’ minimal essential medium (EMEM)-based cell culture medium (Supplementary Material) in 96-well plates and incubated for 24 h in standard conditions. Medium was replaced (60 μL/well) and test compounds (GRM-01 or prednisolone, for preparation see Supplementary Material) or dexamethasone control were added to wells for final assay concentrations of 10 μM–0.003 μM and 10 μM to 0.001 nM, respectively, both in 0.5% DMSO (quadruplicate per test concentration), for a 24-h incubation in standard conditions. Medium was removed, cells washed with 50 μL of phosphate buffered saline (PBS) solution and lysis buffer added (45 µL/well) for a 20-min incubation in agitation at 1,200 rpm at room temperature. Detection buffer (150 µL/well) was added for a 2-h incubation after which the reaction was stopped, followed by a 30-min incubation, all at 37°C. This reaction solution was transferred to an absorbance detection plate (5 µL/well) to measure TAT expression (Envision plate reader, PerkinElmer). For GRM-01 and prednisolone, percentage activation of TAT was calculated (Supplementary Material), and the percentage efficacy and EC_50_ values calculated as described for the inhibition of TNF-α–induced IL-6 release assay, above.

#### Effect on OPG release in human osteoblast cell line

The human osteoblast cell line MG-63 (CRL-1427™, ATCC) was selected to test the effect of GRM-01 on OPG release because MG-63 cells have been used previously to demonstrate the effect of glucocorticoids (and dissociated GR ligands) on OPG release and the reversal of the effect in the presence of the selective GR antagonist Mifepriston (RU486) indicates that the mechanism is driven via GR and does not involve MR. (Humphrey et al., 2006). MG-63 cells cultured in EMEM with 10% FCS were seeded at 3,000 cells/well on 96-well plates and incubated for 24 h in standard conditions, after which the medium was replaced (180 μL) and cells stimulated by adding 20 μL/well of each GRM-01 or prednisolone (triplicates per dilution) for a final assay concentration range of 10 μM to 1 pM in 0.1% DMSO. After a 24-h incubation in standard conditions, supernatant was collected and OPG measured using the human osteoprotegerin/TNFRSF11B DuoSet^®^ ELISA (R&D Systems, Minneapolis, MN, United States), according to the manufacturer’s instructions (Supplementary Material). A 3-parameter nonlinear regression was performed using GraphPad PRISM v7.03 software (Graphpad Software Inc.) to determine IC_50_ values. Data were normalized (the bottom value of the inhibition curve was defined as 0% inhibition and the top value as 100% inhibition).

### *In vivo* pharmacodynamics

#### Efficacy and effect on biomarkers in a rat model of inflammation

Female Lewis rats (150–200 g, Charles River, United Kingdom) were used for the model of joint inflammation (see Supplementary Material for animal care), with *in vivo* efficacy assays conducted at the Saretius Ltd., Science and Technology Centre (Reading, United Kingdom). Monoarticular joint inflammation was induced on Day −21 by intra-articular injection of 10 μg of streptococcal cell wall (SCW) extract (Lee Laboratories, BD Scientific, Franklin Lakes, NJ, United States) into the left hind ankle joint. Intra-articular injections were made under anesthesia, induced using gaseous isoflurane (5% isoflurane/95% oxygen; Merial Animal Health Ltd., Harlow, United Kingdom). On Day −20, rats were allocated to one of seven treatment groups, in groups of 10: GRM-01 0.01, 0.03, 0.1, 0.3, and 1 mg/kg; prednisolone 30 mg/kg; or control (vehicle alone: 0.5% Tween 80 in 1% hydroxypropyl methyl cellulose [HPMC]). Treatments, including control, were administered orally once daily on Days −1 to 6 of the study. Additionally, on Day 0, each rat received an intravenous (IV) dose of 100 μg SCW extract 2 h after oral dosing. IV injections were given after rats had spent up to 10 min in a 40°C hot box (Thermacage MK2, Datesand Ltd., Manchester, United Kingdom).

Rat ankle diameters were measured using digital calipers (Mitutoyo, United Kingdom, Model: CDS6CX), mechanical allodynia tested using von Frey hairs, and rats weighed, per the schedule in Supplementary Table S2. Blood samples were taken on Days 5 and 6 (serial samples 2.5, 4, 6, and 24 h after dosing, and a terminal sample, respectively). On Day 6, rats were euthanized by a schedule 1 method (carbon dioxide) and terminal blood samples collected by cardiac puncture. After centrifugation to obtain plasma supernatant (10,000 rpm, 3 min), plasma was stored at −20°C until analysis. Corticosterone was measured using an ELISA kit (antibody 108,821) from abcam (Cambridge, United Kingdom), following the manufacturer’s protocols, with one exception (plasma samples were diluted 1:50). Glucose test strips (Roche Accu-Check with Aviva blood glucose system meter) were used to measure blood glucose levels at the time blood was drawn. All data were analyzed with Statistica v11 software (StatSoft Inc., Tulsa, OK, United States), using the Kolmogorov-Smirnov test to assess normality of distribution (see Supplementary Material for further detail), and to determine total area under the concentration-time curve (AUC) using the trapezoidal method. Changes in AUC were determined for ankle diameter and mechanical allodynia, between Days −1 to 6, and Days 1 and 5, respectively. The effect of each treatment on ankle inflammation was determined by the reduction in ipsilateral ankle AUC versus control.

#### Effect of GRM-01 on plasma cortisol levels in cynomolgus monkeys

Plasma cortisol levels are suppressed following exogenous glucocorticoid administration through negative feedback mechanisms on the HPA axis (Baker, 2021; Sundahl et al., 2015) and can, therefore, serve as an *in vivo* target engagement biomarker. An exploratory, non-blinded pharmacokinetic/pharmacodynamic (PK/PD) study was therefore conducted, by DMPK Group, ChemPartner Co., Ltd. (Shanghai, China), to investigate the effect of GRM-01 on plasma cortisol levels in male cynomolgus monkeys (*Macaca fascicularis* Raffles*)* following nasogastric administration of single doses (1 mg/kg and 10 mg/kg) to two independent groups of three animals. For animal care, see the Supplementary Material.

GRM-01 was formulated as a homogeneous suspension in 1% HPMC and 0.5% Tween 80 in water and given at a single-dose volume of 5 mL/kg. Blood samples (500 μL each) were taken from animals under restraint, from the cephalic or saphenous veins into K2EDTA tubes at 96 and 48 h pre-dose (for baseline cortisol values), and at pre-dose and 0.25, 0.5, 1, 2, 4, 8, 12, 24, 48, 96, 144, 192, 240, 288, 336, 384, 432, and 480 h post dose. Plasma was obtained within 15 min of sampling by centrifugation (2,000 g, 4°C, 10 min) and stored at approximately −80°C until analysis. GRM-01 and cortisol levels were determined via a validated liquid chromatography with tandem mass spectrometry (LC-MS/MS) method.

A PK/PD evaluation of the data was performed using an indirect response model with a drug inhibition effect on the cortisol production rate by a simple E_max_ model. The concentration-time profile of GRM-01 was fitted using a 1-compartment oral model as supported by the dataset. PK/PD modeling was performed using Phoenix NLME v8.1 (Pharsight Corp., a Certara company, Princeton, NJ, United States).

### Preclinical pharmacokinetics

The single-dose PK of GRM-01 after IV and oral administration was determined by the DMPK Group, ChemPartner Co., Ltd., (Shanghai, China) in female CD-1 mice (n = 9/group), male Sprague Dawley rats (n = 3/group), male and female Beagle dogs (n = 2/group [one of each sex per group]), and male cynomolgus monkeys (*M. fascicularis* Raffles; n = 3/group). The Supplementary Material provides details on the animals and their care.

GRM-01 was administered at a dose of 1 mg/kg for the IV route and 10 mg/kg for the oral route in mice and rats; respective doses were 0.1 mg/kg and 1 mg/kg in dogs, and 1 mg/kg and 10 mg/kg in monkeys. GRM-01 was formulated for IV administration in 5% DMSO + 5% Cremophor EL + 90% dextrose solution (5% of dextrose in water) in the mouse and rat studies, and in 5% DMSO + Kolliphor HS15 + 90% dextrose solution (5% of dextrose in water) in the dog and monkey studies, and for oral administration in 1% hydroxypropyl methylcellulose (HPMC) and 0.5% Tween 80 in water (homogeneous suspension).

IV and oral route administration of GRM-01 was via tail vein injection and oral gavage, respectively, in mice; foot dorsal vein injection and oral gavage, respectively, in rats; and via cephalic vein injection and oral gavage, respectively, in dogs and monkeys. The IV dose was given regardless of the animal’s fed/fasted state in the mouse, rat, dog and monkey PK studies. The oral dose was given regardless of the fed/fasted state in the mouse and rat PK studies, but was given in the fasted state in the dog and monkey PK studies (i.e., animals were fasted overnight and the oral dose given in the morning; fasted animals were fed 4 h after dosing).

Blood samples were collected from each animal under restraint into K2EDTA tubes from mice (∼110 μL/sample) via retroorbital puncture (for sampling pre-dose through to 4 h post dose) or cardiac puncture (for terminal bleeding samples [8, 24, and 36 h post dose]); see Supplementary Material for further detail), from rats (∼150 μL/sample) via tail vein or cardiac puncture, from dogs (∼500 μL/sample) via the cephalic vein, and from monkeys (∼500 μL/sample) via the cephalic or saphenous vein. Samples were collected pre-dose and at 0.083, 0.25, 0.5, 1, 4, 8, and 24 h post dose in all animal species, and additionally at 36 h post dose in mice; 2, 28, 32, and 48 h post dose in rats; 2, 36, 48, and 72 h post dose in dogs; and 2, 48, 72, 96, 120, 144, and 168 h post dose in monkeys. A maximum of three samples per animal was taken in the mouse PK study. To obtain plasma, samples were centrifuged for 5–10 min at 2000 g and 4°C, within 15 min of sampling. Plasma samples were stored at approximately −70°C until analysis using LC-MS/MS.

### Predicted pharmacokinetics in humans

The PK of GRM-01 in humans was predicted based on an integrated analysis of animal *in vivo* concentration-time data using a non-linear mixed effect modeling approach and allometric scaling. The data analysis was performed using NONMEM software (version 7.4; ICON Development Solutions, Hanover, MD, United States), supplemented with the PsN toolkit (version 3.6.2) (Lindbom et al., 2005).

The PK data used in the model were collected from four different animal species (mouse, rat, dog, and monkey) and eight different preclinical studies, where GRM-01 was given as single or multiple doses via the IV or oral routes. Data from four key PK studies in mice, rats, dogs, and cynomolgus monkeys are reported in detail in this manuscript (see ‘Preclinical pharmacokinetics’).

### Data summary and analyses

Unless otherwise stated, continuous variables were summarized and are reported as mean values with standard deviation (SD). Where appropriate, additional statistical analyses were performed as stated in the Figure legends and in the Supplementary Material.

## Results

### *In vitro* pharmacology

#### Nuclear hormone receptor binding and reporter gene assays

In *in vitro* binding assays, GRM-01 was shown to have high selectivity for the GR, given its high affinity for the GR but low affinity for the PR and very low affinity for the MR (Table 1). At the MR, GRM-01 demonstrated a very weak interaction with only 14% inhibition of radioligand ^3^H-aldosterone binding, such that K_i_ and IC_50_ values could not be determined. Similar to GRM-01, prednisolone demonstrated high affinity for the GR and weak binding to the PR (12.1%–30.7% inhibition of ^3^H-progesterone binding). These data indicate that GRM-01, in contrast to prednisolone, is highly selective against MR and therefore has a clearly differentiated binding profile.

**TABLE 1:** Summary of nuclear hormone receptor binding results^a^.

	Glucocorticoid receptor		Progesterone receptor		Mineralocorticoid receptor	
	K_i_, nM	IC_50_, nM	K_i_, nM	IC_50_, nM	K_i_, nM	IC_50_, nM
GRM-01 (n = 2)^b^	12	24	3,700	4,600	ND	>10,000^c^
Prednisolone (n = 2)^b^	5.4	11	ND	>10,000^d^	0.26	0.78

^a^ When percentage inhibition of radioligand binding at a receptor was low, K_i_ and IC_50_ values were not able to be determined.

^b^ n represents the number of replicates.

^c^ Based on 14% inhibition at 10 µM GRM-01.

^d^ Based on no inhibition at 10 µM prednisolone.

IC_50_, half-maximal inhibitory concentration; K_i_, inhibition constant; ND, not determined.

In *in vitro* functional reporter gene assays, GRM-01 produced transactivation at the GR receptor (mean activation = 31.8 ± 3.5%; EC_50_ = 60.2 ± 32.8 nM). GRM-01 did not produce detectable transactivation of either the PR or the MR up to concentrations of 10 µM. In contrast, prednisolone produced transactivation at both the GR (mean activation = 80.5 ± 2.2%; EC_50_ = 24.3 ± 0.5 nM) and the MR (mean activation = 66.4 ± 4.7%; EC_50_ = 11.8 ± 3.2nM), with no detectable transactivation of the PR. At the GR, the ratio of the mean activation level to EC_50_ value was ∼6-fold lower for GRM-01 versus prednisolone, reflecting the overall weaker transactivation potential of GRM-01 in this cellular assay.

#### Inhibition of LPS-induced TNF-α release in rat whole blood assays

The anti-inflammatory potential of GRM-01 was initially determined in an *ex vivo* rat whole blood assay using LPS-induced TNF-α release as the endpoint. GRM-01 displayed an almost complete inhibition of LPS-induced TNF-α release of 96% ± 4%, and an IC_50_ of 79 ± 15 nM (Table 2). In the same assay, prednisolone produced inhibition and IC_50_ values of 99% ± 3% and 8.1 ± 2.1 nM, respectively. When corrected for PPB, the IC_50,free_ values for GRM-01 and prednisolone were 2.5 ± 0.47 nM and 4.7 ± 1.2 nM, respectively, indicating essentially equivalent potency in the whole blood assay.

**TABLE 2:** Summary of key *in vitro* pharmacodynamic properties of GRM-01.

*In vitro* assays	Potency IC_50_/EC_50_ (nM)^a^		Inhibition/Efficacy (%)	
	GRM-01	Prednisolone	GRM-01	Prednisolone
Inhibition of LPS-induced TNF-α release in rat whole blood	n = 8	n = 2	n = 8	n = 2
	79 ± 15^b^	8.1 ± 2.1^b^	96 ± 4	99 ± 3
Inhibition of LPS-induced IFN-γ release in human whole blood	n = 8	n = 8	n = 8	n = 8
	238 ± 225^c^	13.4 ± 7.95^c^	97.2 ± 3.95	99.3 ± 2.8
Inhibition of IL-1β–induced IL-6 release in A549 cells	n = 18	n = 3	n = 18	n = 3
	6.1 ± 2.9	0.86 ± 0.13	93 ± 3.8	101 ± 2.7
Inhibition of TNF-α–induced IL-6 release from FLS from RA patients	n = 3	n = 3	n = 3	n = 3
	19.1 ± 12.0	5.6 ± 2.3	62.8 ± 8.8	79.8 ± 7.6
Induction of TAT enzyme activity in human hepatocyte cell line	n = 6	n = 6	n = 4	n = 4
	>10,000^a^	283.5 ± 113.0^a^	14.0 ± 6.4	92.4 ± 5.3
Inhibition of OPG release in human osteoblast cell line	n = 4	n = 4	n = 4	n = 4
	33 ± 54	3.1 ± 4.3	58 ± 16	100 ± 0

Data are mean ± SD., the n values given for each assay and drug refers to the number of replicates.

^a^ The IC_50_ for GRM-01, and prednisolone was determined in each assay except for the TAT, assay, which determined the EC_50_.

^b^ The IC_50,free_ value was 2.5 ± 0.47 nM for GRM-01, and 4.7 ± 1.2 nM for prednisolone. The IC_50,free_ was calculated as follows: IC_50_ (in whole blood) x fu.

^c^ The IC_50,free_ for was 13.6 ± 12.8 nM for GRM-01, and 3.1 ± 1.8 nM for prednisolone. See footnote b. for the formula for IC_50,free_.

EC_50_, half-maximal effective concentration; FLS, fibroblast-like synoviocytes; fu, fraction unbound; IC_50_, half-maximal inhibitory concentration; IC_50,free_, IC_50_ corrected for plasma protein binding in whole blood; IFN, interferon; IL, interleukin; LPS, lipopolysaccharide; OPG, osteoprotegerin; RA, rheumatoid arthritis; SD, standard deviation; TAT, tyrosine aminotransferase; TNF, tumor necrosis factor.

#### Inhibition of LPS-induced IFN-γ release in human whole blood

In human whole blood *ex vivo* assays, GRM-01 displayed an almost complete inhibition of the LPS-induced IFN-γ release of 97.2% ± 4.0% and an IC_50_ of 238 ± 225 nM (Table 2; Supplementary Figure S1). In the same assay, prednisolone produced inhibition and IC_50_ values of 99.3% ± 2.8% and 13.4 ± 8.0 nM, respectively. When corrected for PPB, the IC_50,free_ values for GRM-01 and prednisolone were 13.6 ± 12.8 nM and 3.1 ± 1.8 nM, respectively. Although, there was greater assay variability versus rat whole blood, GRM-01 displayed a low nM potency in human whole blood.

#### Inhibition of IL-1β–induced IL-6 release in A549 cells

GRM-01 strongly inhibited IL-1β–induced release of IL-6 from immortalized A549 cells by 93% ± 3.8% with an IC_50_ of 6.1 ± 2.9 nM (Table 2). In the same assay, prednisolone produced inhibition and IC_50_ values of 101% ± 2.7% and 0.86 ± 0.13 nM, respectively.

#### Inhibition of TNF-α–induced IL-6 release from primary FLS from patients with rheumatoid arthritis

In cultures of patient-derived FLS, GRM-01 partially inhibited the TNF-α–induced release of IL-6 from primary FLS cells by 62.8% ± 8.8% with an IC_50_ of 19.1 ± 12.0 nM (Table 2; Supplementary Table S3 for individual data). In the same assay, prednisolone also partially inhibited the TNF-α–induced IL-6 release by 79.8% ± 7.6%, with an IC_50_ values of 5.6 ± 2.3 nM.

#### Effect on TAT activity in a human hepatocyte cell line

The *in vitro* gluconeogenic potential of GRM-01 was examined in cultures of the human hepatoma cell line, HepG2, where the induction of TAT enzyme activity was determined. Treatment with GRM-01 resulted in a partial induction of TAT activity to a maximum level of 14.0% ± 6.4% at 10 µM. In contrast, prednisolone, produced a full, concentration-dependent induction of TAT activity to levels of 92.4% ± 5.3% and an EC_50_ value of 283.5 ± 113.0 nM (Table 2).

#### Effect on OPG release in human osteoblast cell line

GRM-01 inhibited OPG release in the immortalized human osteoblast cell line MG-63 in a concentration dependent manner to a maximal level of 58% ± 16% and a potency of 33 ± 54 nM (Supplementary Figure S2). In contrast, prednisolone showed a stronger effect in this assay, fully inhibiting OPG release with a potency of 3.1 ± 4.3 nM (Table 2).

### *In vivo* pharmacodynamics

#### Efficacy and effect on biomarkers in a rat model of inflammation

**FIGURE 1:**
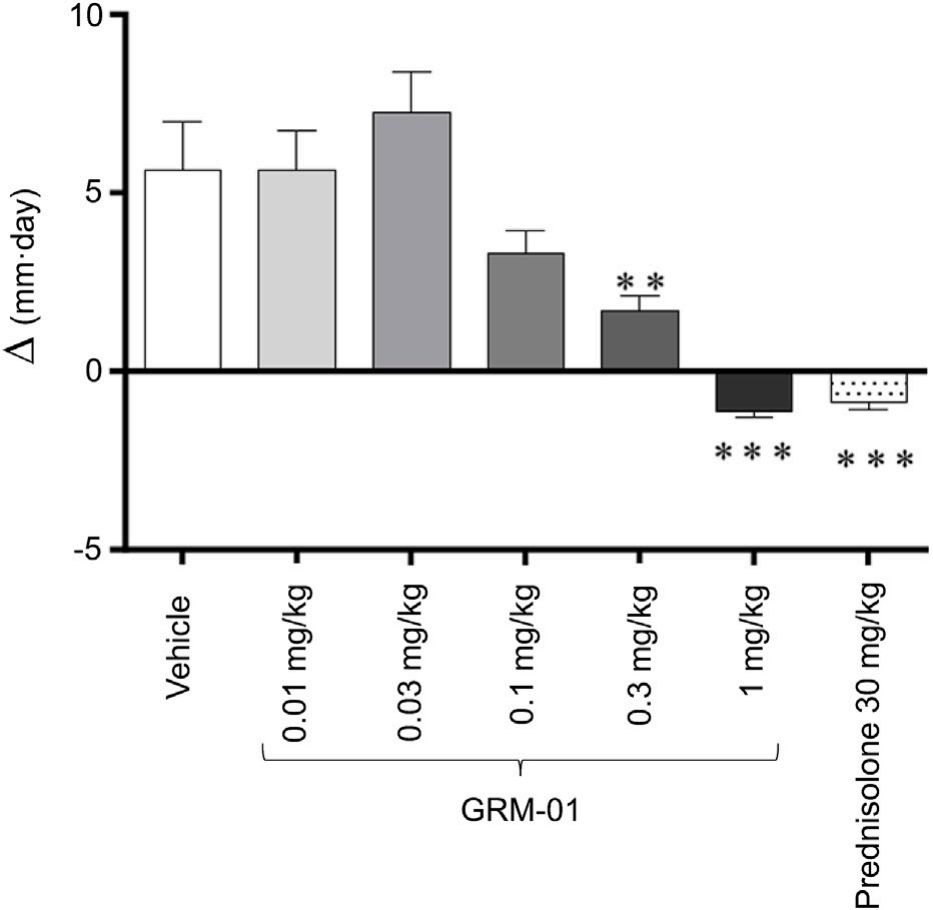
GRM-01 displayed anti-inflammatory effects in the rat SCW model. Changes in the ipsilateral (SCW-injected) ankle diameter over study Days −1 to 6 were expressed as an area under the curve (∆, mm per day) for each treatment group of 10 animals. Treatment was administered daily from Day −1 to 6. Data are mean values with error bars indicating standard error of the mean. **p < 0.01, ***p < 0.001 versus vehicle (control) [Dunn’s multiple comparisons test following significant Kruskal–Wallis analysis of variance]. SCW, streptococcal wall.

The *in vivo* pharmacology of GRM-01 was investigated in the rat SCW challenge model, which is characterized by inflammatory effects on the affected ankle joint that result in ankle swelling. Supplementary Figure S3 shows the increase in ankle joint size induced by the SCW challenge model (see vehicle control group). GRM-01 significantly reduced ankle swelling at doses of 0.3 and 1 mg/kg (p < 0.01 and p < 0.001 vs. control, respectively), according to the change in ipsilateral ankle diameter AUC between Days −1 to 6, as shown in Figure 1. The calculated ED_50_ for GRM-01 was 0.20 mg/kg (95% confidence interval [CI] 0.065–0.619 mg/kg; Supplementary Figure S4). This observed dose-dependent reduction in ankle swelling by GRM-01 reflected its strong, *in vivo* anti-inflammatory activity, which at 1 mg/kg/day was equivalent to 30 mg/kg/day of prednisolone. Administration of the 1 mg/kg/day dose was stopped on day 3 due to bodyweight loss (Supplementary Material), which is a well-characterized effect of higher-dose glucocorticoid administration in rats (Wood et al., 2018).

An antinociceptive effect was demonstrated by GRM-01 at doses of 0.1, 0.3, and 1 mg/kg/day. Increases in the ipsilateral paw withdrawal threshold between Days 1 and 5 were significantly greater with GRM-01 than with vehicle (p < 0.05 for 0.1 mg/kg dose, p < 0.01 for 0.3 mg/kg dose and p < 0.001 for 1 mg/kg dose; Supplementary Figure S5). The ED_50_ was 0.53 mg/kg (95% CI 0.467–0.609 mg/kg). Prednisolone 30 mg/kg/day also demonstrated an antinociceptive effect.

**FIGURE 2:**
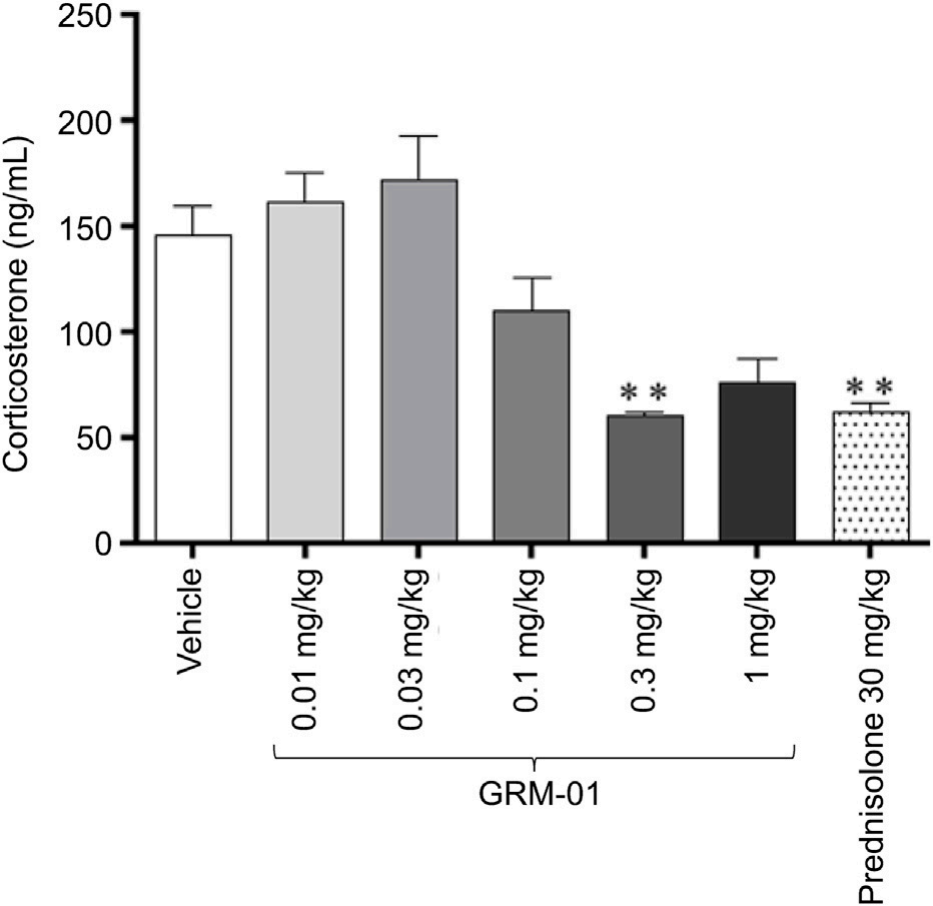
GRM-01 produced dose-dependent reductions in plasma corticosterone levels in the rat SCW model. GRM-01 and prednisolone were administered daily over the treatment period from Day −1 to 6, and corticosterone levels were measured on Day 6. Each treatment group consisted of 10 animals and data are mean values with error bars indicating standard error of the mean. **p < 0.01 versus vehicle (control) based on a Dunn’s multiple comparisons test following a significant result from a Kruskal–Wallis Analysis of variance. SCW, streptococcal wall.

GRM-01 produced dose-dependent reductions in plasma corticosterone levels, which were statistically significant for the 0.3 mg/kg/day dose (p < 0.01 versus vehicle; Figure 2); the GRM-01 ED_50_ was estimated as 0.094 mg/kg (95% CI 0.059–0.15 mg/kg; Supplementary Figure S6). Numerically, GRM-01 at 0.3 and 1 mg/kg/day produced similar reductions in plasma corticosterone levels to prednisolone 30 mg/kg/day, which also reduced plasma corticosterone levels (p < 0.01 versus vehicle).

**FIGURE 3:**
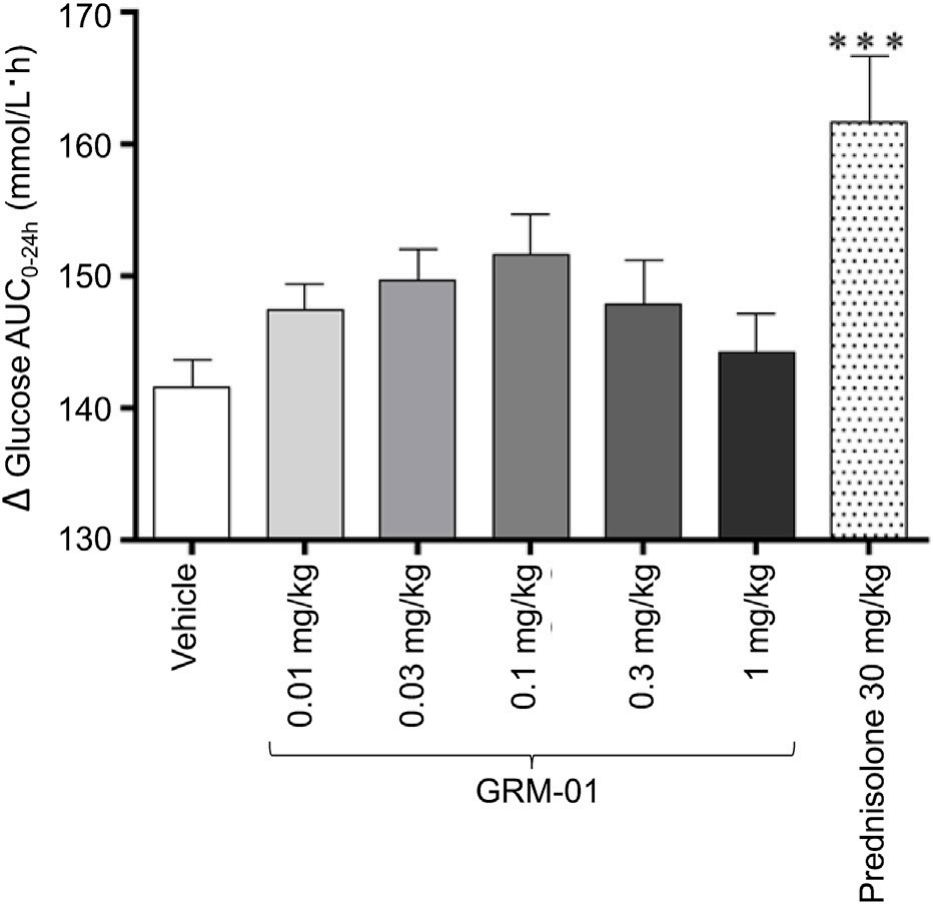
GRM-01 treatment does not produce significant changes in blood glucose area under the curve values in the rat SCW model. Glucose levels were determined on Day 5 over the period 0–24 h (∆AUC0-24h) and the data, 10 animals per treatment group, are shown as mean values with error bars indicating standard error of the mean. ***p < 0.001 versus vehicle (*post hoc* Dunnett’s test following significant one-way analysis of variance test). AUC, area under the curve; SCW, streptococcal wall.

GRM-01 did not produce any significant changes in blood glucose AUC_2.5–24h_ levels versus vehicle at on Day 5 for any dose (Figure 3). In contrast, prednisolone treatment produced a statistically significant increase in blood glucose AUC_2.5–24h_ levels versus vehicle (p < 0.001, Figure 3). Day 5 blood glucose levels at each time point post dose for each dose of GRM-01, and for prednisolone, are shown in Supplementary Figure S7. GRM-01 produced small, statistically significant increases in blood glucose versus vehicle for all doses (p < 0.01 for 0.01 mg/kg/day, p < 0.05 for 0.1 mg/kg/day, p < 0.05 for 0.3 mg/kg/day and p < 0.01 for 1 mg/kg/day), except for the 0.03 mg/kg/day dose at 2.5 h post dose. The increases were not dose dependent. Prednisolone was associated with significantly higher blood glucose levels versus vehicle at 2.5, 4, and 6 h post dose (p < 0.05, p < 0.05 and p < 0.001, respectively).

#### Effect on cortisol as target engagement marker in cynomolgus monkeys

**FIGURE 4:**
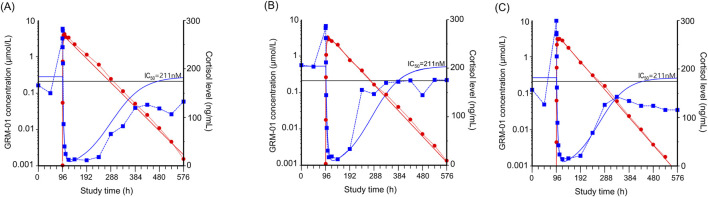
GRM-01 produced an exposure dependent reduction in plasma cortisol levels in male cynomolgus monkeys. Individual data for three animals are presented in **(A–C)** as overlay plots showing the concentration-time profiles of GRM-01 (red circles) and cortisol (blue squares) after administration of a single oral dose of 10 mg/kg at 96 h. The red (GRM-01 concentration) and blue (cortisol level) lines indicate the corresponding pharmacokinetic/pharmacodynamic model fit. The horizontal black line indicates the half-maximal inhibitory concentration (IC50) for cortisol reduction based on the total blood GRM-01 concentrations.

A single oral dose of GRM-01 of 10 mg/kg, which had a mean ± SD maximum plasma concentration (C_max_) of 3.53 ± 0.593 μmol/mL (Supplementary Table S4), strongly suppressed cortisol levels (Figures 4A–C). The cortisol suppression was long lasting, with cortisol levels recovering within 6–12 days, concordant with the plasma concentration-time profile of GRM-01. A single oral dose of GRM-01 1 mg/kg showed only limited effects on cortisol suppression (data not shown). The PK/PD model (Table 3), which used data from both GRM-01 doses, predicted an IC_50_ ± standard error for cortisol suppression by total GRM-01 (bound and unbound) of 211 ± 81 nmol/L (relative standard error [RSE] 38%), and by the unbound fraction of GRM-01 of 10 ± 3.8 nmol/L (RSE 38%). The model predicted full cortisol suppression (maximum inhibitory effect [I_max_] of ∼100%).

**TABLE 3:** Results of the turnover model used to analyze the pharmacokinetic/pharmacodynamic relationship between GRM-01 and cortisol suppression in cynomolgus monkeys.

Parameter	Estimate ± SE (RSE)
tv K_a_ (1/h)	0.329 ± 0.069 (21%)
tv V/f (L/kg)	5.43 ± 0.18 (3%)
tv CL/f (L/kg·h)	0.0947 ± 0.0045 (5%)
tv K_in_ (ng/mL·h)	33.2 ± 6.8 (21%)
tv K_out_ (1/h)	0.190 ± 0.037 (20%)
tv I_max_	1.03 ± 0.04 (4%)
tv IC_50_ (nmol/L)	211 ± 81 (38%)
tv IC_50,free_ (nmol/L)^a^	10 ± 3.8 (38%)

^a^ Fraction of unbound GRM-01, in monkey plasma = 0.0488. The IC_50,free_ was calculated as follows: IC_50_ (in whole blood) x fu.

CL/f, apparent oral clearance; fu, fraction unbound; IC_50_, half-maximal inhibitory concentration; IC_50,free_, IC_50_ corrected for plasma protein binding in whole blood; I_max_, maximum drug-induced inhibition; K_a_, absorption rate constant; K_in_, zero-order turnover rate for production of response; K_out_, fractional turnover rate; RSE, relative standard error; SE, standard error; tv, typical value; V/f, apparent volume of distribution after oral administration.

No abnormal clinical findings were observed in any animals during the study.

### Preclinical pharmacokinetics

GRM-01 demonstrated high oral bioavailability in all four animal species: 76% in mice, 166% in rats, 116% in dogs, and 124% in monkeys, using the standard HBMC/Tween formulation (Table 4). The volume of distribution (V_ss_) was similar in three of the four animal species (1.6–1.9 L/kg in mice, dogs and monkeys), indicating partitioning to tissues. In rats, the V_ss_ was 3.3 L/kg. GRM-01 concentration declined steadily, with a mean terminal elimination half-life (t½) after IV administration of 10.0 h in mice, 12.8 h in rats, 53.4 h in dogs and 34.4 h in monkeys, which was broadly similar to the t½ after oral administration in each of these species (Table 4). Total GRM-01 clearance was generally low after IV administration: 1.4% of liver blood flow (LBF) in mice, 3.8% of LBF in rats, 0.7% of LBF in dogs, and 1.5% of LBF in monkey, which reflects the high metabolic stability of GRM-01 in a range of *in vitro* liver assays across nonclinical species.

**TABLE 4:** The noncompartmental pharmacokinetics of GRM-01 after oral (PO) or intravenous (IV) single-dose administration in four animal species.

Parameter	CD-1 mice		Sprague-Dawley rats		Beagle dogs^a^				Cynomolgus monkeys	
	1 mg/kg, IV (n = 9)	10 mg/kg, PO (n = 9)	1 mg/kg, IV (n = 3)	10 mg/kg, PO (n = 3)	0.1 mg/kg, IV Animal 1	0.1 mg/kg, IV Animal 2	1 mg/kg, PO Animal 3	1 mg/kg, PO Animal 4	1 mg/kg, IV (n = 3)	10 mg/kg, PO (n = 3)
C_0_ (µmol/L)	2.36 ± 0.389	NA	0.998 ± 0.0663	NA	0.156	0.147	NA	NA	1.42 ± 0.209	NA
C_max_ (µmol/L)	NA	7.38 ± 0.809	NA	7.04 ± 1.20	NA	NA	1.13	1.26	NA	11.2 ± 0.794
t_max_ (h)	NA	5.33 ± 2.31	NA	5.33 ± 2.31	NA	NA	8.00	8.00	NA	5.33 ± 2.31
AUC_last_ (h·µmol/mL)	17.8 ± 1.45	134 ± 7.71	11.7 ± 3.41	197 ± 30.9	5.89	5.13	60.3	67.1	53.8 ± 8.12	657 ± 48.3
AUC (h·µmol/mL)	19.4 ± 1.64	148 ± 11.8	13.0 ± 4.14	216 ± 38.2	8.83	9.39	126	122	55.7 ± 8.61	688 ± 50.7
t_1/2_ (h)	10.0 ± 0.566	9.74 ± 1.28	12.8 ± 4.21	11.4 ± 2.50	43.1	63.8	76.1	61.7	34.4 ± 2.83	36.8 ± 1.23
CL (mL/min·kg)	1.81 ± 0.147	NA	2.91 ± 1.03	NA	0.396	0.373	NA	NA	0.640 ± 0.109	NA
CL (%LBF)^b^	1.44 ± 0.117	NA	3.78 ± 1.34	NA	0.708	0.665	NA	NA	1.45 ± 0.247	NA
V_ss_ (L/kg)	1.56 ± 0.0982	NA	3.31 ± 0.652	NA	1.55	2.04	NA	NA	1.87 ± 0.253	NA
F (%)	NA	75.9 ± 6.08	NA	166 ± 29.4	NA	NA	109	122	NA	124 ± 9.11

Data are mean ± SD, except for PK, parameters for dogs which are presented for individual animals. In the column headings, (n = x) refers to the number of animals from which the mean value was obtained.

^a^ One male dog and one female dog per dose group.

^b^ LBF, is 126 mL/min/kg in mice, 77 mL/min/kg in rats, 56 mL/min/kg in Beagle dogs, and 44 mL/min/kg in cynomolgus monkeys.

AUC, area under the concentration-time curve up to infinity; AUC_last_, area under the concentration-time curve up to the last quantifiable concentration; C_0_, calculated concentration at time zero; CL, clearance; C_max_, maximum plasma concentration; F, oral bioavailability; LBF, liver blood flow; n, number of animals; NA, not applicable; PK, pharmacokinetic; SD, standard deviation; t_max_, time to maximum concentration; t_1/2_, terminal half-life; V_ss_, apparent volume of distribution at equilibrium.

There were no notable safety findings in any of the PK studies (Supplementary Material).

### Predicted pharmacokinetics in humans

The PK of GRM-01 were best described by a 2-compartment model with linear absorption and linear elimination. The exponents of the allometric scaling in the final allometric model were identical to the theoretical value of 0.75 for clearance and inter-compartmental clearance, and a theoretical value of 1 for the V_ss_. The predicted total plasma clearance was 0.25 mL/min/kg, corresponding to 1.05 L/h in a 70-kg human, with a V_ss_ of 2.124 L/kg (corresponding to 149 L in a 70-kg human). The predicted t_½_ in humans was ∼98 h.

## Discussion

The studies reported herein demonstrate that GRM-01 is a novel potent selective GR modulator, with both *in vitro* and *in vivo* preclinical models confirming its anti-inflammatory efficacy, and *in vitro* cell-based models suggesting limited effects on bone formation and gluconeogenesis. Our results suggest a preclinical profile for GRM-01 that potentially dissociates the beneficial anti-inflammatory effects of GR activation from clinically-relevant adverse effects typically associated with chronic corticosteroid treatment.

*In vitro*, GRM-01 demonstrated potent GR binding affinity (K_i_ = 12 nM) that was highly selective, with a ∼300-fold higher affinity for the GR over the PR. GRM-01’s binding affinity for the MR was at least ∼5000-fold lower than that of prednisolone, suggesting that GRM-01 shows stronger selectivity than prednisolone for the GR over the MR. Results of nuclear hormone receptor reporter gene assays indicated that GRM-01 exhibited less GR transactivation than prednisolone, a pharmacologic feature that, in theory, may improve the adverse event profile of GRM-01 in the clinical setting (Ripa et al., 2018). The potential therapeutic effect of GRM-01 was demonstrated in assays in whole blood (rat and human) and in human cells (A549 cell line and primary synovioctyes *ex vivo* from patients with rheumatoid arthritis). GRM-01 displayed prednisolone-like suppression of LPS-induced TNF-α release from rat whole blood, LPS-induced IFN-γ release from human whole blood, and IL-6 release from A549 cells and synoviocytes. Compared to the full agnostic efficacy of prednisolone, the *in vitro* anti-inflammatory effects of GRM-01 appear to be more pronounced than those observed for the GR-selective partial agonist dagrocorat (PF-00251802, PF802) (Hu et al., 2011), which is the active moiety of the administered drug fosdagrocorat (PF-04171327) (Ripp et al., 2018). Prednisolone displayed 100% inhibition of IL-6 release in A549 cells in the dagrocorat study (Hu et al., 2011), and 101% in our A549 assay, whereas dagrocorat produced 78% inhibition (Hu et al., 2011), and in our assay GRM-01 produced 93% inhibition. When inhibition of IFN-γ release in human whole blood was examined, dagrocorat produced ∼60–70% inhibition versus ∼85–100% inhibition by prednisolone (at concentrations each of 100–1000 nM) (Hu et al., 2011), whereas GRM-01 gave 97% inhibition versus 99% by prednisolone in this study.

GRM-01 displayed *in vivo* evidence of target engagement and robust anti-inflammatory efficacy in preclinical animal models. In the rodent SCW *in vivo* model of ankle inflammation, GRM-01 produced dose-dependent decreases in plasma corticosterone levels, confirming target engagement through activation of the negative feedback loop in the HPA axis by drug bound GR reducing endogenous glucocorticoid cortisol production (Sundahl et al., 2015). This was also evident in cynomolgus monkeys receiving a single oral dose of GRM-01 (10 mg/kg) resulting in a strong suppression of plasma cortisol levels. The model estimated an IC_50,free_ value of ∼10 nM, with full cortisol suppression (as seen with prednisolone), indicating high target engagement. GRM-01 also produced a strong anti-inflammatory efficacy signal in the SCW model, reducing ankle swelling with an ED_50_ dose of 0.2 mg/kg, and additionally, increased the time to paw withdrawal due to mechanical stimulation with von Frey hairs (ED_50_ dose of 0.5 mg/kg), indicative of an antinociceptive behavioral effect. GRM-01 (1 mg/kg/day) was as effective as prednisolone (30 mg/kg/day) in reducing ankle swelling, and more effective than prednisolone (30 mg/kg/day) in increasing the time to paw withdrawal.

*In vitro* models of glucose metabolism and bone loss demonstrated clear differentiation of GRM-01 from prednisolone at concentrations that had produced an anti-inflammatory effect. Activation of GR target gene activity in the gluconeogenesis pathway in the liver is a key mechanism in the development of hyperglycemia, an established adverse effect of exogenous glucocorticoid treatment that can lead to diabetes mellitus (Li and Cummins, 2022). In a human hepatocyte cell line, GRM-01 had a minimal effect on the activity of TAT, a key enzyme in gluconeogenesis (Panin and Usynin, 2008), with only a 14% induction in activity at the highest tested GRM-01 concentration of 10 μM, compared with full activity (92%) induced by prednisolone (EC_50_ = 283 nM). Notably, results from the *in vivo* rat model of inflammation supported these *in vitro* findings, with no significant effect on blood glucose AUC_2.5–24h_ levels on Day 5 in animals treated with GRM-01 (versus vehicle), whereas prednisolone produced a statistically significant increase in this endpoint.

One of the deleterious effects of glucocorticoid treatment is the inhibition of bone formation and thus bone homeostasis. This is mediated, in part, via an inhibitory effect on osteoblast differentiation and activity (van Staa, 2006). Osteoblasts produce OPG, which prevents osteoclastogenic activity by binding to RANKL (van Staa, 2006). It has been previously shown that glucocorticoids decrease OPG expression in a concentration-dependent manner in human cell culture (Sivagurunathan et al., 2005). In the human osteoblast MG-63 cell line, GRM-01 had an ∼2-fold reduced effect, versus prednisolone, on OPG release suggesting a differentiated and more favorable profile on an important bone homeostasis marker.

GRM-01 showed high oral absorption, with a slow and similar plasma clearance after IV or oral administration in four preclinical PK studies (in mice, rats, dogs, and cynomolgus monkeys). The decline in GRM-01 plasma concentration was similar after IV or oral administration in these four studies. Based on an allometric population PK analysis using the aforementioned preclinical PK data from different animal species (as well as other preclinical PK data, not reported here), a long t_½_ of ∼98 h was predicted for humans. This is much longer than t_½_ values reported for prednisolone (∼2.5 h) (Ferry et al., 1988) or mizacorat (∼5 h) (Ripa et al., 2018).

Based on the positive preclinical and PK findings included in this report, GRM-01 has been investigated in healthy volunteers in a single-ascending dose trial, as well as a multiple-dose ascending trial (data on file).

Some study limitations should be acknowledged. Firstly, characterization of the metabolic effects of GRM-01 and prednisolone was conducted only on cell lines (e.g., HepG2 and MG-63), which, although established models, may not fully re-capitulate compound effects on primary cells or *in vivo* pathways. Secondly, the mapping of GRM-01’s effects on metabolic pathways was targeted to key genes only, and this does not capture the full range of transcription changes. It is anticipated that these gaps will be addressed in future clinically-focussed research activities. Finally, it is important to acknowledge that the outcomes from preclinical *in vivo* models may not translate into the clinic. For example, in glucocorticoid induced osteoporosis (GIOP) there is a lack of guidelines for applying available animal models so that they can accurately predict outcomes in humans (Xavier et al., 2021). This was, in part, mitigated by the use of an established inflammatory rat model that focused on quantifiable outcomes that are not susceptible to animal behavior. Allowing for these points, the overall and extensive preclinical data reported herein supports further development of GRM-01 as a novel therapeutic agent for a range of inflammatory diseases.

## Conclusion

In summary, *in vitro* and *in vivo* models confirmed the anti-inflammatory effects of GRM-01, demonstrating clear differentiation from prednisolone, with no effect on glucose levels in an *in vivo* rat model of inflammation, and limited inhibition of OPG release from human osteoblasts *in vitro*. These results indicate that by selectively binding to the GR receptor, GRM-01 triggers anti-inflammatory effects but with the potential advantage of having less severe adverse effects on glucose levels and bone metabolism than glucocorticoids. Animal PK data suggested a favorable profile for oral administration of GRM-01, and results from the allometric population PK analysis predicted a long half-life of GRM-01 in humans.

Future research is required to investigate the benefit-to-risk ratio of GRM-01 in patients with inflammatory diseases, and confirm whether the long-term treatment hurdles typically associated with glucocorticoids have been overcome. In addition, it will be interesting to investigate whether the differentiation from prednisolone observed here with GRM-01 is also of value in other clinical diseases or disorders where glucocorticoids remain a cornerstone of treatment.

## Data Availability

The original contributions presented in the study are included in the article/Supplementary Material, further inquiries can be directed to the corresponding author.
