# Supplementary material for: Novel selective glucocorticoid receptor modulator GRM-01 demonstrates dissociation of anti-inflammatory effects from adverse effects on glucose and bone metabolism
Source: Front Pharmacol. 2025 Mar 5;16:1542351. doi: 10.3389/fphar.2025.1542351 (PMC11920646; doi:10.3389/fphar.2025.1542351)
Supplement: Supplementary file 1 [file DataSheet1.docx]

Supplementary Material

# Supplementary Methods

## *In vitro* pharmacology

### Nuclear hormone receptor binding

Results of binding assays were expressed as a percent of control specific binding, calculated as follows:

$\frac{measured specific binding}{control specific binding} ⨯100$

Percent inhibition of control specific binding was calculated as follows:

$$100-\left( \frac{measured specific binding}{control specific binding}⨯100 \right)$$

The half-maximal inhibitory concentration (IC_50_; i.e., the concentration causing a half-maximal inhibition of control specific binding) and Hill coefficients (n_H_) were determined by non-linear regression analysis of the competition curves generated with mean replicate values using the following Hill equation curve fitting:
$Y=D+ \left[ \frac{A-D}{1+ \left( C/C_{50} \right)^{nH}} \right]$ , where Y = specific binding, A = left asymptote of the curve, D = right asymptote of the curve, C = compound concentration, C50 = IC_50_, and nH = slope factor.

The inhibition constants (K_i_) were calculated using the Cheng Prusoff equation:

K_i_=IC_50_/(1+L/K_D_)

where L = concentration of radioligand employed in the assay, and K_D_ = affinity of the radioligand for the receptor (a Scatchard plot was used to determine the K_D_) (Cheng and Prusoff, 1973).

### Nuclear hormone receptor reporter gene assays

Transactivation was calculated according to this equation:

$$\% activation= \left( \frac{\left( compound -min \right)}{\left( \max- min \right)} \right)\times100$$

where min = the assay buffer–only value and max = the value for the reference agonist (beclomethasone, progesterone, or fludrocortisone).

### Rat lipopolysaccharide (LPS)/tumor necrosis factor-alpha (TNF-α) whole blood assay

For the blood stimulation protocol, GRM-01 and prednisolone were diluted 1:4 in 100% dimethyl sulfoxide (DMSO) starting from a 10 mM stock for generation of a 10-point dilution series, and then further diluted 1:100 in medium (RPMI medium [Thermo FisherScientific, Darmstadt, Germany], 10% fetal calf serum [FCS], without DMSO).

#### TNF-α analysis protocol

In the commercially available mouse TNFalpha Kit (PerkinElmer), anti-TNF-α antibodies reveal >80% cross-reactivity for rats and can therefore also be used to examine rat plasma samples. The immuno-assay-buffer provided in the kit was diluted 1:10 in water and the mouse TNF-α analyte standard was diluted in rat plasma (30,000–4 pg/mL). Each standard dilution or plasma sample (2.5 μL each) were added to the wells of a 384-well polystyrene compound plate, to which 10 µL of the test mix 1 (containing AlphaLISA anti-TNF-α acceptor beads and biotinylated anti-TNF-α antibody diluted 1:200 in immuno-buffer) was added to each well and incubated for 1 h at room temperature. Afterwards, streptavidin donor beads were diluted 1:62.5 in immuno-assay-buffer and 12.5 µL were transferred to each well. After a 30-min incubation at room temperature, the plates were read using an EnVision Multilabel Reader® (PerkinElmer) equipped with the ALPHA option using the AlphaScreen standard settings.

#### Calculation of IC_50,free_

The following formula was used to determine the IC_50,free_ value:

IC_50,free_ = IC_50_ (in whole blood) x fu

Where fu = fraction unbound.

### Human LPS/interferon (IFN)-γ whole blood assay

A 6-point 1:5 dilution series of each of GRM-01 and prednisolone were prepared for this assay in 100% DMSO, reconstituted to a 30 mM stock solution that was stored at −20°C, which was then used to prepare the solutions (using 100% DMSO) for use in the whole blood assay at the following concentrations: 0.0096, 0.048, 0.24, 1.2, and 6 mM.

### Interleukin (IL)-6 release assay in a human lung cell line

GRM-01 and prednisolone were prepared from a 10 mM stock to generate an 8-point dilution series and then further diluted 1:200 in fetal calf serum (FCS)-free F12K nutrient mix.

#### Immunoassay protocol

Per the human AlphaLISA® (PerkinElmer) immunoassay kit instructions, the immuno-assay buffer was diluted 1:10 in water and the human IL-6 analyte standard was diluted in FCS-free F12K nutrient mix (30,000–10 pg/mL). Each standard dilution or sample (5 μL each) was added to the wells of a 96-well polystyrene compound plate, to which 20 µL of the test mix 1 (containing anti-IL-6 acceptor beads and biotinylated anti-IL-6 antibody diluted 1:200 in immuno-assay-buffer) was added to each well and incubated for 1 h at room temperature. Afterwards, donor beads were diluted 1:62.5 in immuno-assay-buffer and 25 µL were transferred to each well. After a 30-min incubation at room temperature, the plates were read using an EnVision Multilabel Reader® (PerkinElmer) equipped with the ALPHA option.

### IL-6 release by primary fibroblast-like synoviocytes (FLS) from patients with rheumatoid arthritis

For this assay, GRM-01 and prednisolone were prepared to generate a 10-point log dilution curve, starting at a concentration of 10 mM with a 100% DMSO stock solution, giving a final assay concentration range for testing of 10 μM to 0.1 pM (final DMSO concentration 0.1%).

#### Data analysis

The relative inhibition of IL-6 release by GRM-01 or prednisolone was calculated using the following equation:

$$\% inhibition= \left( \left( compound -max \right)/\left( \min- max \right) \right) \times100$$

where min = IL-6 secretion in medium only, max = maximum IL-6 secretion after stimulation with TNF-α at 90% maximal effective concentration (EC_90_).

### Effect on tyrosine aminotransferase (TAT) activity in human hepatocyte cell line

The HepG2 cell culture medium was comprised of 500 mL of Eagles’ minimal essential medium (EMEM) without L-glutamine, 5 mL of 100X penicillin/streptomycin, 5 mL of Ultraglutamine I Supplement 200 mM, 5 mL of non-essential amino acids (NEAA) 100X (all from Lonza), and 50 mL of FBS (Sigma).

Solutions of GRM-01, prednisolone, and dexamethasone (the reference agonist) were prepared to generate an 8-point half-log compound dilution curve, starting from a 2 mM stock solution in 100% DMSO, and then diluted 1:50 in culture medium.

#### Data analysis

The percentage activation of TAT expression by GRM-01 and prednisolone was calculated according to the following equation using Genedata Screener® version 15.0.5 software (Genedata, Basel, Switzerland):

$$\% activation= \left( \left( compound -min \right)/\left( \max- min \right) \right) \times100$$

where min = TAT expression in medium only, max = TAT expression after stimulation with dexamethasone

### Effect on osteoprotegerin (OPG) release in human osteoblast cell line MG-63

#### Eight-point serial dilutions of GRM-01 and prednisolone were prepared for this assay from 10 mM stock and diluted 1:100 in complete cell culture medium (EMEM + 10% FCS).

#### ELISA assay protocol

All incubations in this assay were conducted at room temperature. Anti-OPG capture antibodies, diluted in Dulbecco's phosphate-buffered saline (DPBS), were transferred into a coated 96-well plate (100 µL/well). After overnight incubation, and triplicate washing (400 µL/well) with wash buffer (0.05% Tween 20 in DPBS, pH 7.2–7.4), 300 µL of Reagent Diluent (1% bovine serum albumin [BSA] in DPBS) was added, followed by a 1-h incubation, and thereafter three washing steps again. Then, 100 µL of supernatant, standard or reagent diluent were added per well for a 2-h incubation. After repeating three washing steps, 100 µL of detection antibody diluted in reagent diluent (including normal goat serum) were added to each well, followed by a 2-h incubation. After repeating three washing steps, 100 µL of Streptavidin conjugated to horseradish-peroxidase solution was added per well for a 20-min incubation. Finally, 50 µL/well of stop solution was added. After gently shaking of the plate to ensure thorough mixing, the optical density of each well was determined immediately using the Eon™ Microplate Spectrophotometer (Bio Tek Instruments) set at 450 nm.

## *In vivo* pharmacodynamics

### Efficacy and effect on biomarkers in a rat model of inflammation

#### Animal care and housing

Rats were housed under standardized conditions (12-h light/dark cycles, 19–22°C, 45–65% relative humidity), in accordance with UK Home Office regulations, in groups of six in Tecniplast 1354 cages (Tecniplast UK, London, UK) with standard softwood bedding (Litaspen 8/20, Lillico, Horley, UK) and environmental enrichment (Envirodri/cardboard tunnels, Lillico, UK). Rats had free access to pelleted standard laboratory chow (RM3 expanded diet, Special Diet Services Ltd, Witham, UK) and tap water in bottles. When wet mashed diet was required, RM3E was used.

#### Statistical methods

Data that was not normally distributed were analyzed with Kruskal-Wallis analysis of variance (ANOVA) followed by Dunn’s multiple comparisons tests. Normally distributed data were analyzed with repeated measures ANOVAs as appropriate, one-way ANOVAs and Dunnett’s post hoc tests.

### Effect on cortisol as target engagement marker in cynomolgus monkeys

#### Animals and animal care

Six male cynomolgus monkeys (4.6–5.6 kg) were obtained from Hainan Jingang Biotechnology Co. Ltd. A certified non-human primate diet was provided to all animals twice a day with water available *ad libitum*. Animals were fasted overnight prior to GRM-01 administration, and fed 4-h post dose on study days. In addition to the animals to whom GRM-01 was administered, one additional animal that did not receive GRM-01 was used for the collection of blood (5–10 mL) to be used as a blank sample for the development of the analytical method.

## Preclinical pharmacokinetics

### Animals and animal care

Eighteen female CD1 mice, each weighing 30­–32 g, were obtained from Beijing Vital River Laboratory Animal Co. Ltd for the pharmacokinetic (PK) study. Animals had free access to food and water. Retroorbital blood samples for PK sampling from predose to 4 h post dose were collected under anesthesia with 2–3% isoflurane and oxygen administered at a flow rate of 0.5–0.8 L/min. Blood samples for PK analysis at 8, 24 and 36 h post dose were collected from terminal bleeding via cardiac puncture under euthanasia with pure CO_2_ via inhalation. Death was confirmed by a veterinarian checking the heartbeat and respiration.

Six male Sprague Dawley rats, each weighing 20–260 g, were obtained from Shanghai Jihui Laboratory Animal Co. Ltd for the PK study. Animals had free access to food and water.

Four Beagle dogs (2 male, 2 female; 7.24–8.60 kg) were obtained from Beijing Marshall Biotechnology Co. Ltd for the PK study. Animals assigned to receive GRM-01 via intravenous administration had free access to food and water, whereas those assigned to oral GRM-01 were fasted overnight (prior to dosing) and were fed only 4 hours post dose, although access to water was ad libitum throughout.

Six male cynomolgus monkeys were obtained from Hainan Jingang Biotechnology Co. Ltd. (3.22–4.22 kg), as well as one additional animal. Food and water were supplied using the same schedule as for dogs based on route of GRM-01 administration.

# Supplementary Results

## *In vivo* pharmacodynamics

### Rat model of inflammation

A dose-dependent weight loss was observed in the GRM-01 group, with the dose required to produce a response that was 50% percent of the maximum possible effect (ED_50_) being 0.15 mg/kg (95% confidence interval [CI] 0.084–0.272 mg/kg). Dosing was therefore stopped early in the 1 mg/kg GRM-01 group, with the last dose administered on day 3. Bodyweight loss is known to occur in rats after high-dose glucocorticoid administration (Wood et al., 2018).

## Preclinical pharmacokinetics

There were no abnormal clinical effects of GRM-01 administration observed in any animals in the PK studies, although a male dog experienced a small amount of loose stool ~8 h post an oral GRM-01 dose.

# Supplementary Figures and Tables

## Supplementary Figures

**Supplementary Figure S1.** Concentration-response curves of GRM-01 and prednisolone on LPS-induced IFN-γ release in human whole blood (n=10). Compound concentration is plotted on the x-axis on a logarithmic scale. IFN-γ indicates percentage inhibition, calculated relative to the LPS-stimulated vehicle. Data are mean values, error bars depict the standard deviation. IFN-γ , interferon gamma; LPS, lipopolysaccharide.


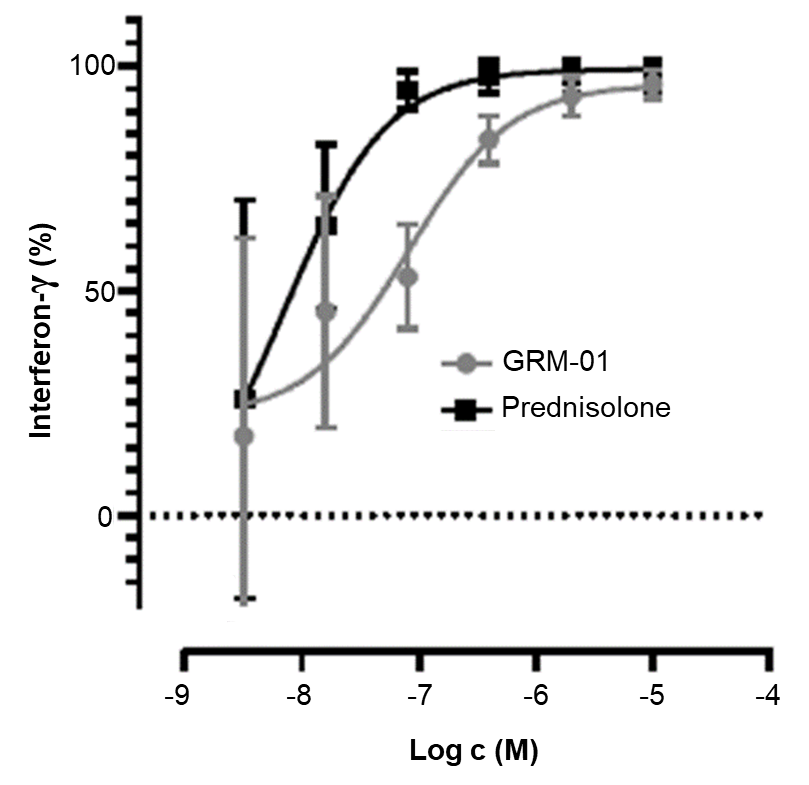


**Supplementary Figure S2.** Concentration-response curves of GRM-01 and prednisolone on osteoprotegerin (OPG) release in MG-63 human osteoblast cells. Data are mean values from 4 independent experiments with GRM-01 and 3 with prednisolone. Error bars indicate standard error of the mean. Data were normalized by setting prednisolone inhibition at 100%. GRM-01 and prednisolone concentrations were 0.000001, 0.00001, 0.0001, 0.001, 0.01, 0.1, 1, and 10 μM, shown in a logarithmic scale on the x-axis. Log c, log concentration.


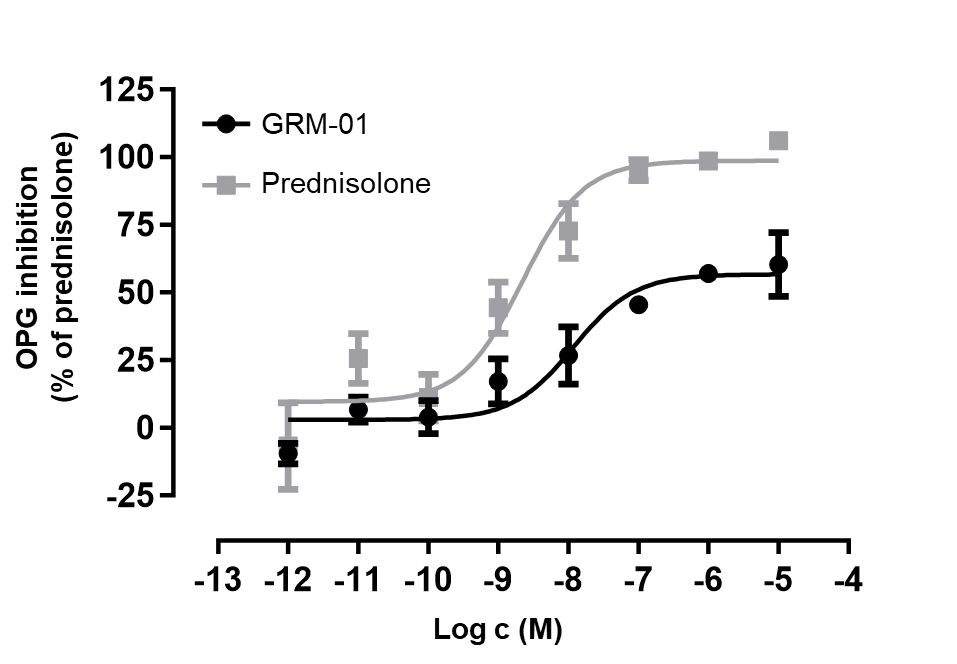


**Supplementary Figure S3.** The effects of GRM-01 (0.01, 0.03, 0.1, 0.3, and 1 mg/kg) and prednisolone (30 mg/kg) on streptococcal cell wall-induced model of ankle inflammation in rats (10 animals per dose). Data are mean values with error bars indicating standard error of the mean (10 animals per dose). ∆, change in ankle diameter from Day −1.


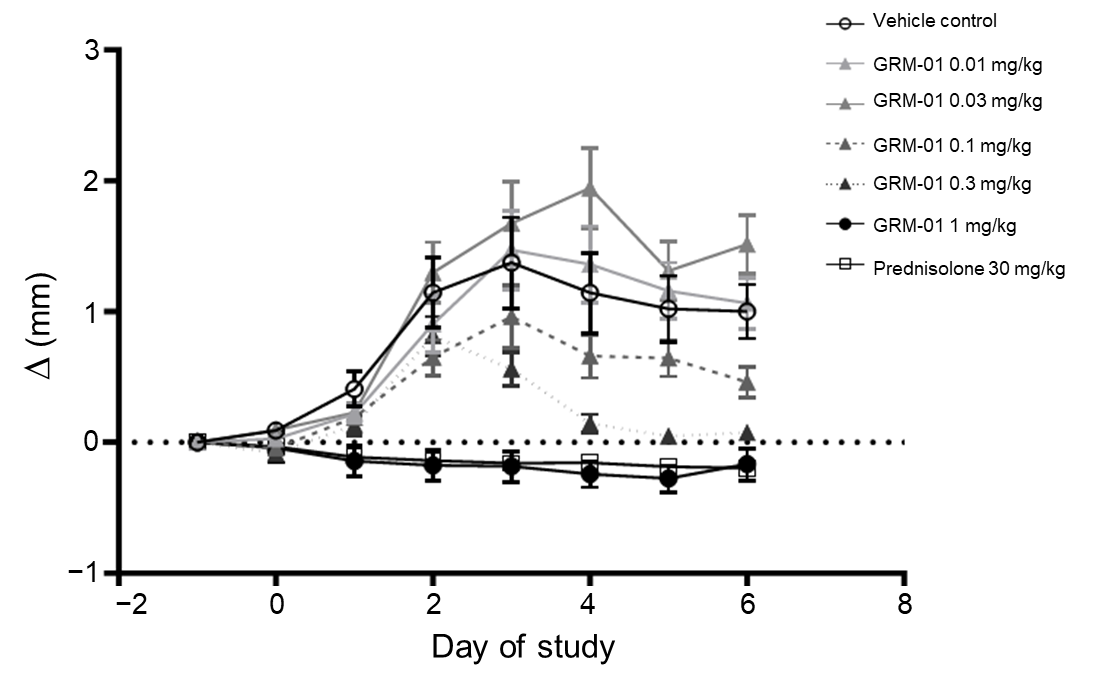


**Supplementary Figure S4.** GRM-01 dose-response curve for the change in area under the concentration-time curve (∆) of the ankle diameter of rats with streptococcal cell wall-induced inflammation. GRM-01 doses were 0.01, 0.03, 0.1, 0.3, and 1 mg/kg, shown in a logarithmic scale on the x-axis. Data are mean values with error bars indicating standard error of the mean (10 animals per dose). Based on curve fitting, with no constraints and an R value of 0.5651, the GRM-01 ED_50_ was determined to be 0.20 mg/g (95% confidence interval: 0.065–0.619). ED_50_, dose required to achieve a response 50% of the maximum possible effect.


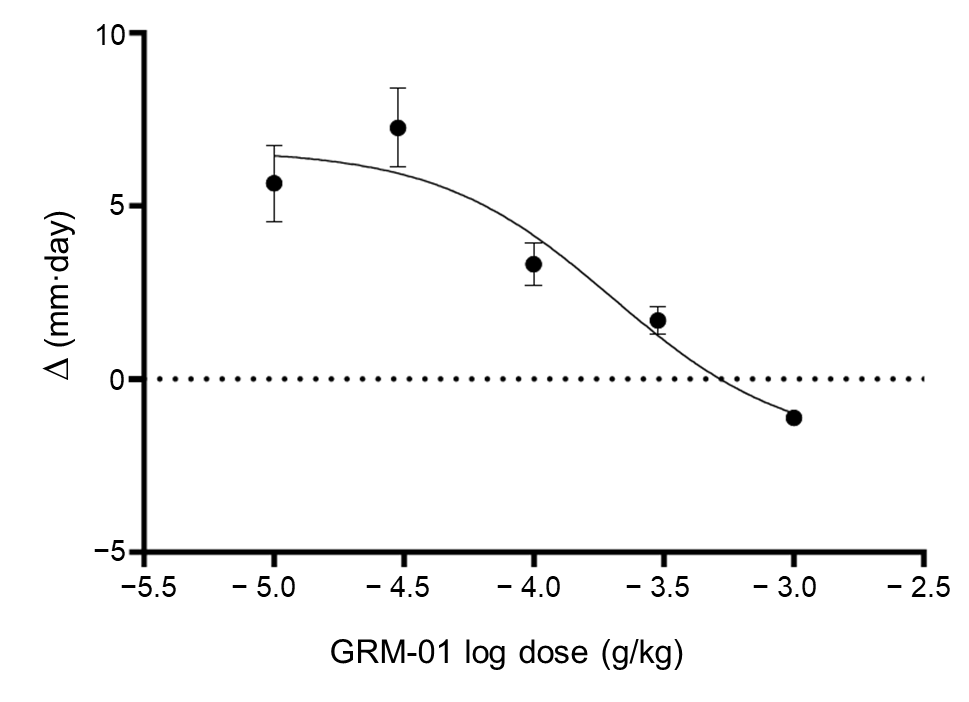


**Supplementary Figure S5.** The effect of GRM-01 (0.01, 0.03, 0.1, 0.3, and 1 mg/kg) and prednisolone (30 mg/kg) on mechanical allodynia in the ipsilateral paw in a streptococcal cell wall-induced model of inflammation in rats (10 animals per treatment group). Animals received GRM-01 or prednisolone daily between Days −1 and 5. Mechanical allodynia was assessed prior to ankle measurements on Days −1, 1, 3, and 5. Data are mean AUC for paw withdrawal threshold between Days 1 and 5, with error bars indicating standard error of the mean. * p<0.05, ** p<0.01, and ***p<0.001 versus vehicle control (post hoc Dunnett’s test following significant one-way analysis of variance test). AUC, area under the concentration curve.


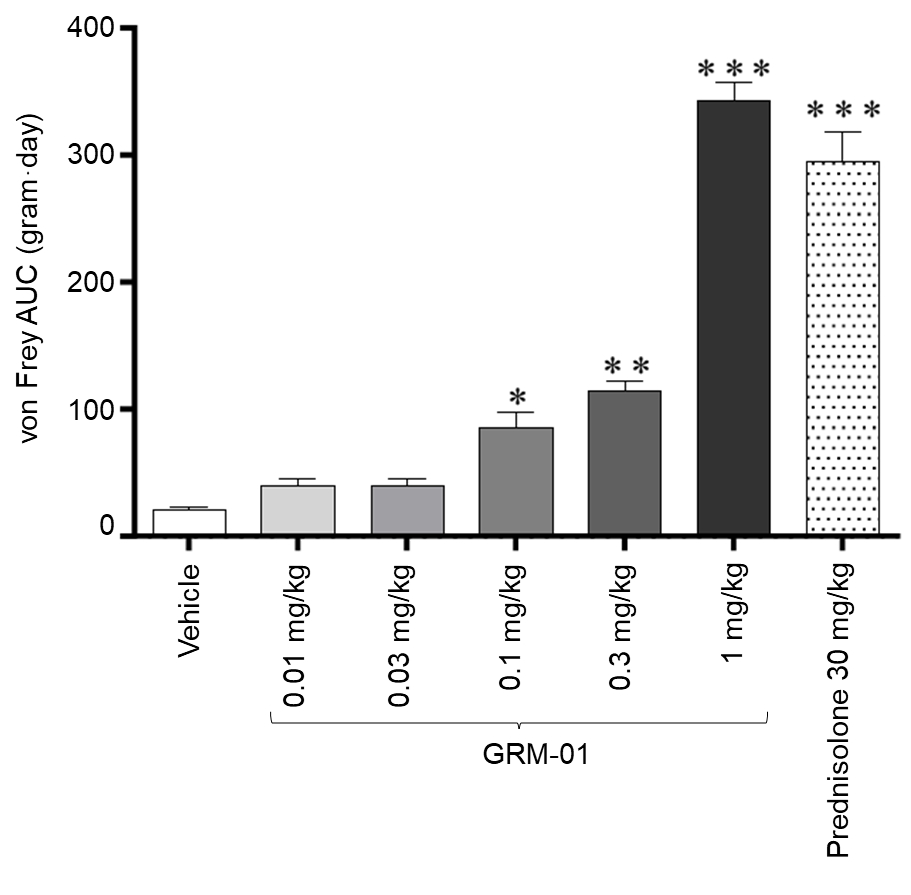


**Supplementary Figure S6.** GRM-01 dose-response curve for plasma corticosterone levels on Day 6 in rats with streptococcal cell wall-induced inflammation of the ankle. GRM-01 doses were 0.01, 0.03, 0.1, 0.3, and 1 mg/kg, shown in a logarithmic scale on the x-axis. Data are mean values with error bars indicating standard error of the mean (10 animals per dose). Based on curve fitting, with constraints (top = 180, bottom = 50) and an R square value of 0.4886, the GRM-01 ED_50_ was calculated as 0.09 mg/kg (95% confidence interval: 0.059–0.150). ED_50_, dose required to achieve a response 50% of the maximum possible effect.


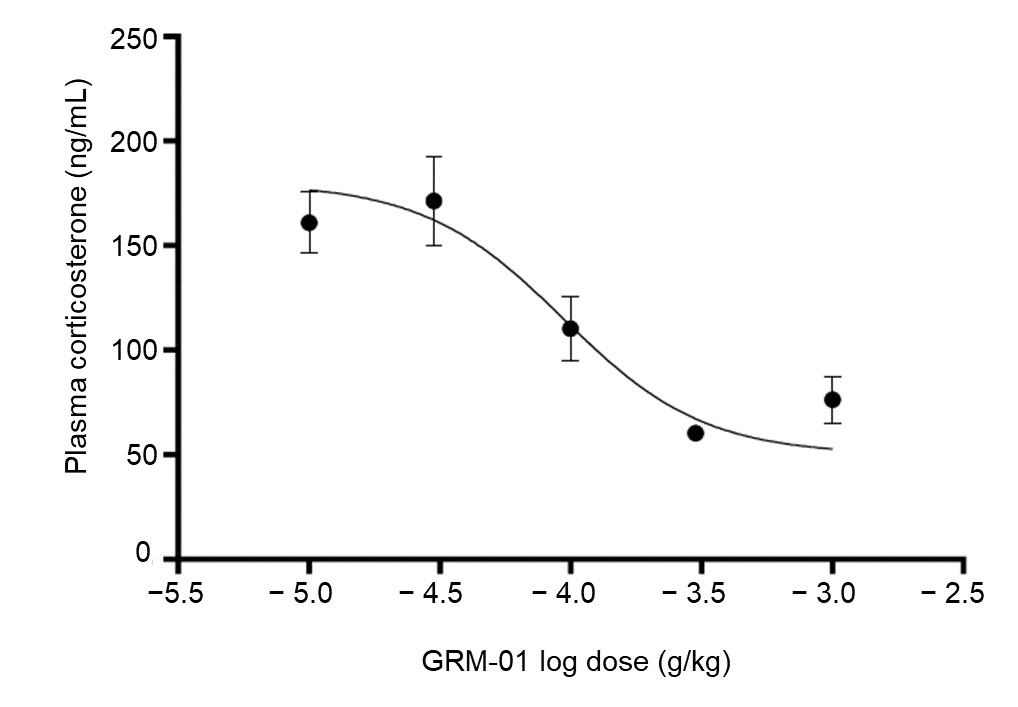


**Supplementary Figure S7.** The effect of GRM-01 (0.01, 0.03, 0.1, 0.3, and 1 mg/kg) and prednisolone (30 mg/kg) on blood glucose concentration in a streptococcal cell wall-induced model of inflammation in rats (10 animals per treatment group). Blood samples for glucose measurement were collected 2.5, 4, 6 and 24 h after dosing on Day 5. Data are mean values with error bars indicating standard error of the mean. * p<0.05, **p<0.01, *** p<0.001 versus vehicle control (post hoc Dunnett’s test following significant one-way analysis of variance test).


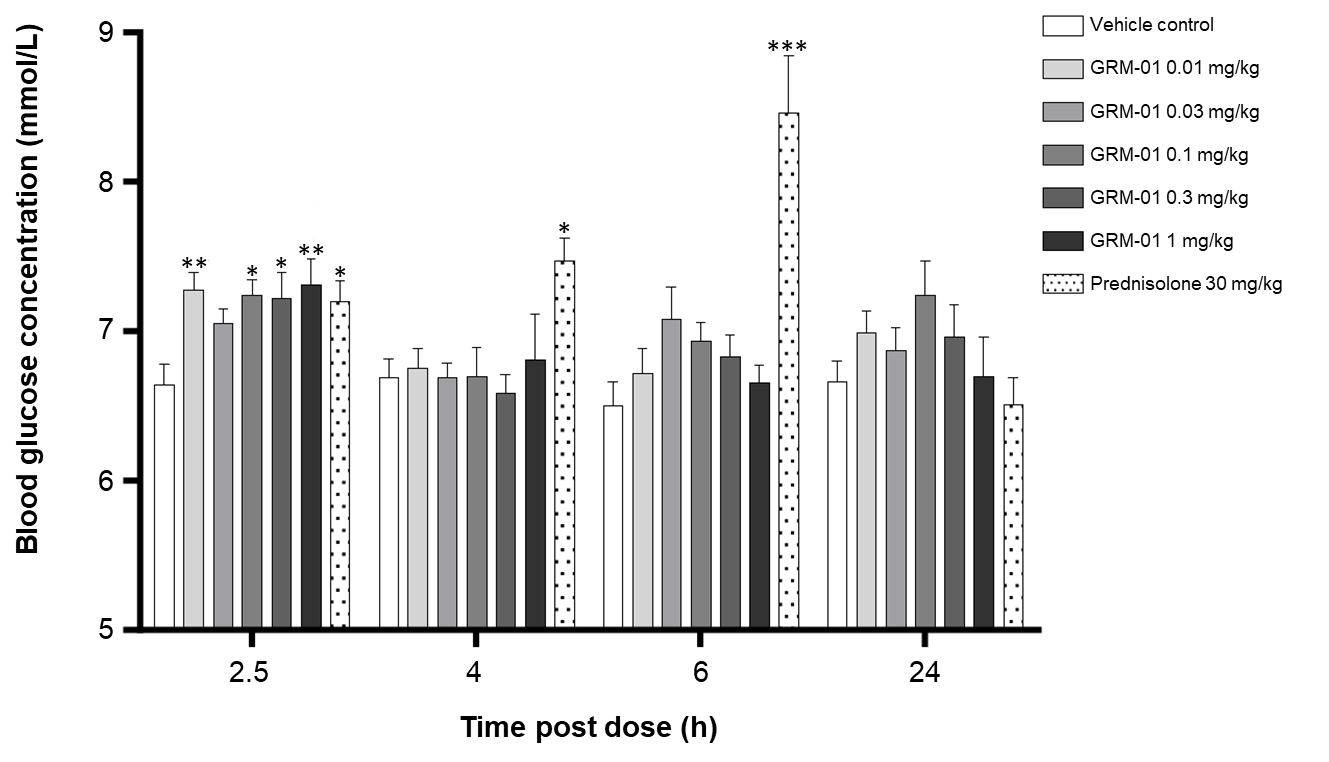


**Supplementary Figure S8.** Concentration-response curves of GRM-01 and prednisolone in reporter gene induction (transactivation) assays of human (A) GR, (B) PR, and (C) MR in a CHO cell line overexpressing human GR, PR, or MR ligand binding domain (LBD) fused to the GAL4 DNA binding domain (LBD-DBD). Data are mean values from 4-8 independent experiments with GRM-01 and 3 with prednisolone. Error bars indicate standard error of the mean. GRM-01 and prednisolone concentrations were 0.003, 0.01, 0.03, 0.1, 0.3, 1, 3, and 10 μM, shown in a logarithmic scale on the x-axis. Log c, log concentration.


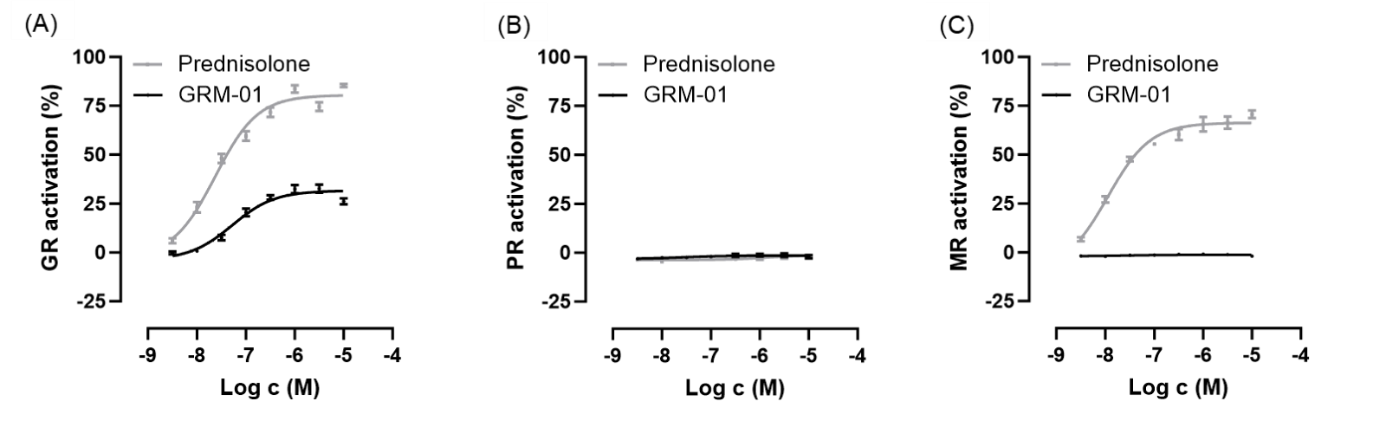


**Supplementary Figure S9** Prednisolone dose-response curve (from separate and independent study) for the change in area under the concentration-time curve (∆) of the ankle diameter of rats with streptococcal cell wall-induced inflammation. Prednisolone doses were 0.003, 0.03, 0.3, 3, and 30 mg/kg, shown in a logarithmic scale on the x-axis. Data are mean values with error bars indicating standard error of the mean (10 animals per dose). Based on curve fitting, with no constraints and an R value of 0.5982, the Prednisolone ED_50_ was determined to be 2.49 mg/kg (95% confidence interval: 1.07–5.81). ED_50_, dose required to achieve a response 50% of the maximum possible effect.

**
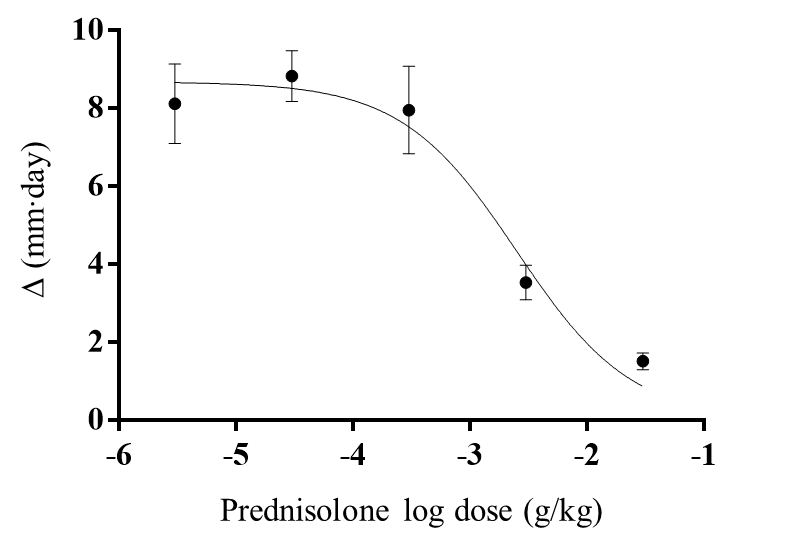
**

**Supplementary Figure S10.** The effect of Prednisolone (0.003, 0.03, 0.3, 3.0, and 30 mg/kg) on blood glucose concentration in a streptococcal cell wall-induced model of inflammation in rats (10 animals per treatment group). Blood samples for glucose measurement were collected 2.5, 4, 6 and 24 h after dosing on Day 5. Data are mean values with error bars indicating standard error of the mean. * p<0.05, **p<0.01, *** p<0.001 versus vehicle control (post hoc Dunnett’s test following significant one-way analysis of variance test).


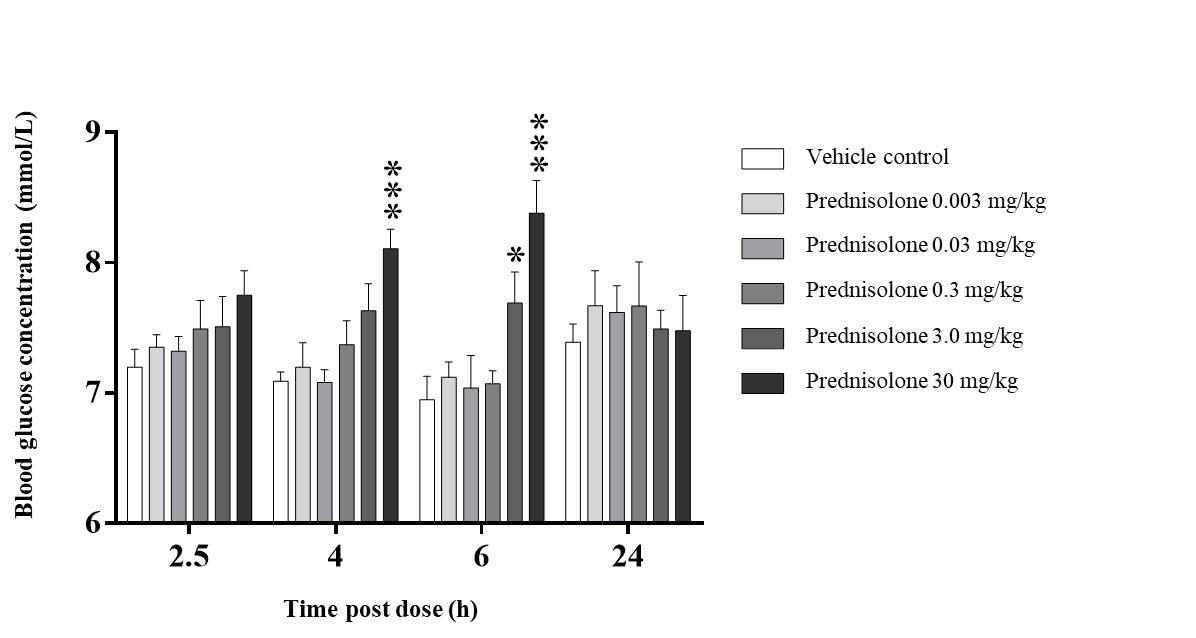


## Supplementary Tables

**Supplementary Table S1.** Interferon (IFN)-γ human whole blood assay procedure

|  | **Assay procedure** | **Setting (per well)** | **Duration** |
| --- | --- | --- | --- |
| 1 | Wash the plate three times with wash buffer | ≥ 300 μL | 3 cycles |
| 2 | Add 50 μL of each prepared standard, QC, blanks, and sample per well | 50 μL | N/A |
| 3 | Cover the plate with adhesive strip and incubate for 2 h at RT on a horizontal microplate shaker at 350 rpm | 350 rpm, RT | 2 h |
| 4 | Wash the plate three times with wash buffer | ≥300 μL | 3 cycles |
| 5 | Add 25 μL of the prepared detection antibody to each well | 25 μL | N/A |
| 6 | Cover the plate with adhesive strip and incubate for 2 h at RT on a horizontal microplate shaker at 350 rpm | 350 rpm, RT | 2 h |
| 7 | Wash the plate three times with wash buffer | ≥300 μL | 3 cycles |
| 8 | Add 150 μL of 2XMSD read buffer to each well | 10 μL | N/A |
| 9 | Read the plate on the MSD instrument | N/A | N/A |

h, hour; MSD, Meso Scale Discovery; N/A, not applicable; rpm, revolution per minute; RT, room temperature; QC, quality control

**Supplementary Table S2.** Study procedures in the *in vivo* pharmacodynamic study in rats

| **Day** | **Events** |
| --- | --- |
| −21 | Measure ankles, ankle SCW injections (n=80) |
| −20 | Measure ankles |
| −8 | VF habituation^a^ |
| −7 | VF habituation^a^ |
| −6 | VF habituation^a^ |
| −1 | VF, measure ankles and body weight, PO dose |
| 0 | Measure ankles and body weight, PO dose, IV dose 2 h later |
| 1 | PO dose, VF and measure ankles and body weight 2 h later |
| 2 | PO dose and measure ankles and body weight 2 h later |
| 3 | PO dose, VF and measure ankles and body weight 2 h later |
| 4 | PO dose and measure ankles and body weight 2 h later |
| 5 | PO dose, VF and measure ankles and body weight 2 h later, and take blood samples and measure glucose at 2.5, 4, 6, and 24 h post dose |
| 6 | 24-h blood sample and measure glucose, PO dose and measure ankles and body weight 2 h later, terminal blood sampling |

^a^Habituation involves repeatedly applying the von Frey hair to the paw to reduce the likelihood that response by the animal is due to a startle effect, rather than pain (Malfait et al., 2013).

h, hour; IV, intravenous; PO, per oral; SCW, streptococcal cell wall; VF, Von frey hairs.

**Supplementary Table S3.** Concentration-dependent GRM-01 and prednisolone inhibition of tumor necrosis factor-α (TNF-α )–induced interleukin-6 (IL-6) release from primary fibroblast-like synoviocytes from three patients with rheumatoid arthritis (Donors 1, 2, and 3)

|  |  | **IL-6 concentration (pg/mL)** | | | | | | | | | |
| --- | --- | --- | --- | --- | --- | --- | --- | --- | --- | --- | --- |
|  |  | **Donor 1** | | | **Donor 2** | | | | **Donor 3** | | |
|  |  | 1 | 2 | 3 | 1 | 2 | 3 | 1 | | 2 | 3 |
|  | **Concentration [log M]** |  |  |  |  |  |  |  | |  |  |
| **GRM-01** | **−14** | 1012.2 | 900.1 | 904.8 | 1543.4 | 1246.1 | 1319.9 | 1071.8 | | 985.0 | 1048.4 |
|  | **−13** | 914.1 | 900.1 | 881.5 | 1389.1 | 1197.1 | 1280.5 | 1020.9 | | 1014.6 | 4273.4^a^ |
|  | **−12** | 918.7 | 881.5 | 900.1 | 1897.5 | 1177.5 | 1114.1 | 997.7 | | 1042.1 | 920.1 |
|  | **−11** | 900.1 | 830.4 | 867.5 | 1324.8 | 1216.7 | 1138.5 | 1110.2 | | 980.8 | 795.9 |
|  | **−10** | 881.5 | 858.3 | 737.9 | 1354.4 | 1017.1 | 1192.2 | 1146.7 | | 997.7 | 1061.2 |
|  | **−9** | 867.5 | 839.7 | 811.9 | 1364.3 | 1085.0 | 1021.9 | 934.7 | | 2508.2^a^ | 924.3 |
|  | **−8** | 742.5 | 761.0 | 664.2 | 1065.5 | 829.5 | 973.6 | 665.7 | | 800.0 | 645.5 |
|  | **−7** | 554.3 | 590.9 | 522.4 | 973.6 | 648.8 | 620.4 | 438.8 | | 399.6 | 466.3 |
|  | **−6** | 431.5 | 508.7 | 413.4 | 658.2 | 535.6 | 535.6 | 273.7 | | 352.9 | 294.9 |
|  | **−5** | 336.6 | 341.1 | 345.6 | 644.0 | 530.9 | 526.2 | 327.7 | | 323.8 | 287.2 |
| **TNF-α control**^b^ | | 928.1 | 918.7 | 951.4 | 1354.4 | 1339.6 | 1206.9 | 1071.8 | | 1239.7 | 1059.0 |
| **Predniso-lone** | **−14** | 1106.3 | 960.2 | 880.6 | 1552.3 | 1393.1 | 1255.1 | 947.8 | | 888.3 | 831.3 |
|  | **−13** | 1101.6 | 838.7 | 755.0 | 2326.4 | 1123.2 | 1142.7 | 1151.3 | | 956.1 | 937.5 |
|  | **−12** | 946.2 | 768.9 | 708.6 | 1427.8 | 1215.9 | 1123.2 | 859.7 | | 904.7 | 837.4 |
|  | **−11** | 964.9 | 801.4 | 731.8 | 1497.4 | 1079.5 | 2570.2 | 796.9 | | 774.8 | 813.1 |
|  | **−10** | 880.6 | 778.2 | 796.8 | 1309.2 | 1108.6 | 1215.9 | 983.0 | | 1022.6 | 1127.9 |
|  | **−9** | 988.4 | 736.4 | 685.5 | 1338.8 | 1045.5 | 1157.3 | 637.6 | | 645.5 | 847.5 |
|  | **−8** | 616.3 | 483.3 | 446.8 | 848.1 | 695.7 | 681.5 | 386.6 | | 621.9 | 428.5 |
|  | **−7** | 292.3 | 274.3 | 238.1 | 554.0 | 394.9 | 404.3 | 214.4 | | 221.8 | 220.0 |
|  | **−6** | 269.7 | 215.6 | 206.6 | 521.2 | 394.9 | 381.0 | 179.4 | | 170.2 | 159.2 |
|  | **−5** | 260.7 | 224.6 | 206.6 | 516.5 | 385.6 | 394.9 | 166.5 | | 172.0 | 199.6 |
| **TNF-α control**^b^ | | 1030.8 | 960.2 | 1358.8 | 1602.4 | 1108.6 | 1527.4 | 1102.5 | | 997.6 | 776.8 |

^a^Experimental outliers; excluded from analysis.

^b^Tested at 90% maximal effective concentration.

**Supplementary Table S4.** The pharmacokinetic parameters of GRM-01 after oral administration of a single dose to male cynomolgus monkeys

|  | **GRM-01 single dose** | |
| --- | --- | --- |
| **Parameter** | **1 mg/kg (n = 3)** | **10 mg/kg (n = 3)** |
| C_max_ (µmol/mL) | 0.423 ± 0.0311 | 3.53 ± 0.593 |
| t_max_ (h) | 6.00 ± 5.29 | 9.33 ± 2.31 |
| AUC_last_ (h·µmol/mL) | 22.4 ± 2.39 | 250 ± 43.9 |
| AUC_∞_ (h·µmol/mL) | 22.5 ± 2.39 | 250 ± 43.9 |
| t_1/2_ (h) | 38.9 ± 2.34 | 40.1 ± 1.83 |

Data are presented as mean ± SD. In column headings, the n value refers to the number of animals.

AUC_∞_, area under the concentration-time curve from time 0 to infinity; AUC_last_, area under the concentration-time curve from time 0 to the last quantifiable concentration; C_max_, maximum plasma concentration; SD, standard deviation; t_max_, time to maximum concentration; t_1/2_, terminal half-life.

# References

Cheng, Y., and Prusoff, W. H. (1973). Relationship between the inhibition constant (K_1_) and the concentration of inhibitor which causes 50 per cent inhibition (I_50_) of an enzymatic reaction. Biochem. Pharmacol. 22, 3099-3108. doi: 10.1016/0006-2952(73)90196-2

Malfait, A. M., Little, C. B., and Mcdougall, J. J. (2013). A commentary on modelling osteoarthritis pain in small animals. Osteoarthritis Cartilage. 21, 1316-1326. doi: <https://doi.org/10.1016/j.joca.2013.06.003>

Wood, C. L., Soucek, O., Wong, S. C., Zaman, F., Farquharson, C., Savendahl, L., et al. (2018). Animal models to explore the effects of glucocorticoids on skeletal growth and structure. J. Endocrinol. 236, R69-R91. doi: 10.1530/JOE-17-0361
